# TimeTeller: A tool to probe the circadian clock as a multigene dynamical system

**DOI:** 10.1371/journal.pcbi.1011779

**Published:** 2024-02-29

**Authors:** Denise Vlachou, Maria Veretennikova, Laura Usselmann, Vadim Vasilyev, Sascha Ott, Georg A. Bjarnason, Robert Dallmann, Francis Levi, David A. Rand

**Affiliations:** 1 Mathematics Institute & Zeeman Institute for Systems Biology and Infectious Disease Epidemiology Research, University of Warwick, Coventry, United Kingdom; 2 Division of Biomedical Sciences, Warwick Medical School, University of Warwick, Coventry, United Kingdom; 3 Odette Cancer Centre, Sunnybrook Health Sciences Centre, Toronto, Ontario, Canada; 4 Department of Statistics, University of Warwick, Coventry, United Kingdom; 5 UPR “Chronotherapy, Cancer and Transplantation”, Medical School, Paris-Saclay University, Medical Oncology Department, Paul Brousse Hospital, Villejuif, France; Pázmány Péter Catholic University: Pazmany Peter Katolikus Egyetem, HUNGARY

## Abstract

Recent studies have established that the circadian clock influences onset, progression and therapeutic outcomes in a number of diseases including cancer and heart diseases. Therefore, there is a need for tools to measure the functional state of the molecular circadian clock and its downstream targets in patients. Moreover, the clock is a multi-dimensional stochastic oscillator and there are few tools for analysing it as a noisy multigene dynamical system. In this paper we consider the methodology behind TimeTeller, a machine learning tool that analyses the clock as a noisy multigene dynamical system and aims to estimate circadian clock function from a single transcriptome by modelling the multi-dimensional state of the clock. We demonstrate its potential for clock systems assessment by applying it to mouse, baboon and human microarray and RNA-seq data and show how to visualise and quantify the global structure of the clock, quantitatively stratify individual transcriptomic samples by clock dysfunction and globally compare clocks across individuals, conditions and tissues thus highlighting its potential relevance for advancing circadian medicine.

## Author summary

The cellular circadian clock consists of an interacting set of genes that through their interactions oscillate throughout the day. This oscillator also responds to external cues so that the genes oscillate in phase with external environmental rhythms. A cell therefore uses its circadian clock to provide its genes with information about the external time. In this way it can coordinate many of the processes taking place in the cell and allocate some of these processes to specific times of the day. It is becoming increasingly clear that the quality of this timing information influences onset progression and outcome in a number of chronic diseases such as cancer. Our aim is therefore to develop a machine-learning tool that can assess how well the clock is working. We want to use this with patients and therefore, for clinical utility, it needs to work with only a single clinical sample and to produce reproducible results that can be clearly interpreted and easily compared.

## Introduction

The mammalian cell-endogenous circadian clock temporally regulates tissue-specific gene expression driving rhythmic daily variation in metabolic, endocrine, and behavioural functions. Indeed, up to half of all mammalian genes are expressed with a circadian rhythm in at least one tissue [1, 2] and approximately 50% of all current drugs target the product of a circadian gene [1]. Moreover, recent studies demonstrated that the circadian clock influences therapeutic outcomes in a number of diseases including heart disease and cancer [3–9], and that disruption of the normal circadian rhythm and sleep (e.g., through shift work) is associated with a higher risk of obesity, hypertension, diabetes, chronic heart disease, stroke and cancer [10–13]. There is therefore a rapidly growing interest in developing circadian medicine tools that aid the incorporation of time in order to provide safer and more efficacious therapeutics.

As a result a number of phase-estimation algorithms have been designed to estimate the molecular clock phase of the circadian clock, i.e., its “internal time”, from the measured levels of rhythmic gene expression [14–22]. If the sample collection time (SCT) is known, then divergence between the estimated timing *T* and the SCT indicate the possible presence of clock dysfunction and, indeed, this internal phase *T* has been proposed as a clinically actionable biomarker [23]. There are problems with such an approach, the most obvious of which is that this internal time may well depend substantially upon genotype or environment (as we show below) and the consequent deviations are unlikely to be related to dysfunction. A different attempt at a systemic approach to define molecular clock disruption has used pair-wise correlations between clock genes across large transcriptomic datasets [24]. At the population level, this showed greater dysfunction in solid tumours compared to healthy tissue. However, this approach compared datasets of cohorts with each other and, as the authors pointed out, does not lend itself to assessing clock function in single samples. A similar approach using clock correlation matrices together with CYCLOPS ordering [18] and a measure called nCV [25] that correlates positively with clock amplitude was used to address clock dysfunction in pancreatic cancer cells [26].

The core mammalian circadian clock involves more than a dozen genes [27] and therefore the regulatory system is a high dimensional stochastic dynamical system. Since emergent systems properties such as oscillation, synchronisation, entrainment, phase-locking, robustness, flexibility and temperature compensation are critical for the functioning of the clock, tools that enable the analysis of the circadian clock’s systems properties are very much needed. Moreover, a substantial amount of data is becoming available including whole transcriptome time-series that should facilitate such systems analysis using mathematical modelling, statistics and machine learning. However, probing the global behaviour of such a system is a highly non-trivial task and almost all analysis of clock data focuses on individual components and connections. This is not the case for the phase estimation algorithms mentioned above but they adopt a model-blind machine-learning approach. While such approaches can be effective it is difficult to see how to quantify clock functionality independently in individual samples without taking advantage of the clock’s structure as a stochastic dynamical system because it is this that determines the well-defined probabilistic structure describing the relationship between time and multidimensional gene state that, via statistical theory, can be linked to functionality.

To effectively quantify functionality in individual transcriptomic samples such as those from patients requires reproducibility, comparability and interpretability. Therefore, the results on a given test sample should be independent of those on other test samples and should not depend upon the particular test dataset being considered. Even for timing estimation alone this does not seem possible with the phase-estimation algorithms mentioned above apart from TimeSignature [19] which requires two samples. However, the key point differentiating TimeTeller from TimeSignature and the other algorithms is that, apart from identifying timing deviations, these do not provide any other assessments of clock functionality or other quality controls on the individual timing assessments. This is essentially also true for ZeitZeiger [15] but with the caveat that it, like TimeTeller, uses a likelihood curve that it might be considered could be used in a similar way to TimeTeller’s to assess functionality. However, although differences in ZeitZeiger’s likelihood between WT/control and perturbed clocks in controlled experimental situations has been discussed [15], it has not been proposed or statistically analysed as a measure of dysfunction and has not been used as such when ZeitZeiger has been employed to analyse timing variation in populations [23, 28, 29]. Moreover, analysis by ZeitZeiger of new data as described in [15] involves renormalizing and batch-correcting this data with the training data and then retraining, resulting in a different predictive model every time and therefore potentially sacrificing the reproducibility, comparability and interpretability discussed above.

Our aim is to develop a tool that (i) provides a multidimensional picture of the clock’s dynamics and structure that integrates the behaviour of multiple genes, (ii) provides a quantitative analysis at the systems level of clock data, (iii) enables a quantitative comparison of different clocks and (iv) enables a quantitative assessment of clock dysfunction both in the core clock and in downstream target genes. We are aiming for a tool that can determine the presence of a dysfunction causing perturbation from just one sample and that can stratify individuals based on clock functionality, and, thus, might be useful to develop as a clinically actionable biomarker. For example, we show that such a stratification can enable the identification of differentially expressed genes between samples that have better and worse clocks. Finally, we consider new methods for comparing clocks across different individuals, tissues and conditions, identifying a “molecular chronotype” associated with these, and uncovering the effect of clock perturbations on downstream genes.

It is important to understand the limits on what we regard as dysfunction in our discussion. TimeTeller’s view of functionality is based on statistical analysis of gene expression and not on timing of physiological processes. The probability structure of the dynamical system behind a circadian clock is primarily described by the joint probability distribution *P*(*t*, *g*) of the external time *t* and the expression state *g* of the core clock genes or some representative function or subset of them. This distribution determines the conditional distributions *P*(*t*|*g*) and *P*(*g*|*t*). These distributions tell us respectively the distribution of *g* when the time is *t* and the probability distribution of times *t* that are found when the gene expression state of the clock is *g*. The distribution *P*(*t*|*g*) is a critical quantity because the cell has to use some function of the state of the gene products as a surrogate for *t* and the variance of *P*(*t*|*g*) tells us how well cells can tell the time by just seeing the clock gene state. If *g* comes from a test sample taken from a well aligned clock with internal time *T* (possibly distant from the SCT) then we would expect that *P*(*g*|*T*) would be relatively large and, as a function of time *t*, *P*(*t*|*g*) would be sharply peaked at *t* = *T*. From the point of view of TimeTeller if either of these breaks down then the sample’s clock is regarded as dysfunctional to some extent. We quantify this breakdown by a measure *ML* of the probability that *g* is drawn from the training clock and another that combines a measure of the variance of the clock’s estimate of the time and a quantity related to the existence of multiple peaks in *P*(*t*|*g*).

It is also important to stress here that with the currently available data we will have to make and justify some assumptions on the cross-validity of data from different tissues in order to combine the data. For example, in order to estimate the probability model for a particular tissue we would ideally like to use training data that is only from that tissue. In particular, this is not possible for the mouse and baboon datasets as adequate amounts are not currently available and we therefore have to pool data from several tissues. To do this we choose an appropriate rhythmic gene panel based on good cross-tissue synchronicity and, after validation of this, use normalisation to overcome tissue differences in the way explained below and in Fig A in S1 Appendix. For our human datasets we pool across individuals rather than tissues. Another potential limitation comes from the fact that our current RNA-seq training data is only available at a few training time points around the day. Nevertheless, even with these handicaps we obtain very informative results and provide plenty of evidence that the approaches adopted work well. As more data becomes available this situation can only improve.

## Results

### Training with genetically homogeneous and heterogeneous data

The broad range of transcriptomes from microarray and RNA-seq data that we use is detailed in Note A in S1 Appendix as are the methods used to prepare the data for use with TimeTeller. The data that is used to prepare TimeTeller’s probability model is referred to as the *training data*. The data that is then analysed using this probability model is called *test data*. In this paper we use four different training datasets and more details about these are in Note A in S1 Appendix.

#### Choice of a clock representative gene panel

For a given training dataset we firstly choose the panel of *G* rhythmic genes that TimeTeller will use. This is called the *rhythmic expression profile* (REP). For a given transcriptomics sample the expression levels *g*_*k*_, *k* = 1, …, *G*, of these genes are collected into a vector *g* = (*g*_1_, …, *g*_*G*_) which we will call the *rhythmic expression vector* (REV). The user is free to choose the genes in the REP and may have a particular reason to include or leave out a particular gene. However, in this study we first carry out an analysis of both the rhythmicity and synchronicity across tissues or individuals in our datasets to guide our choice. This analysis, which is detailed in Fig B in S1 Appendix, is important to choose a panel of genes with good circadian rhythmicity combined with minimal variation across the relevant tissues or individuals and to try to ensure it provides a faithful representation (Note B in S1 Appendix) of the clock state even though it might not contain all core clock genes.

#### Timecourse and intergene normalisation

When combining training data from multiple tissues, for each gene in the REP we study the variation across the tissues in that gene’s expression time-series. This analysis (Fig B in S1 Appendix) shows that for RNA-seq data this variation is significantly greater than that found, for example, in the Affymetrix MoGene 1.0 ST and GeneChip Human Genome U133 Plus 2.0 microarray platforms that we have analysed. Therefore, for the RNA-seq training data, it is usually necessary if we are combining data from multiple tissues to carry out what we call timecourse normalisation.

Each of our training data sets is organised into time series for each gene in the REP with times *t*_*k*_, *k* = 1, …, *K*, that are usually independent of the particular gene. We can normalise the data by replacing each of these time series by a normalised version which has mean expression zero and standard deviation 1. We call this approach *timecourse normalisation*. Following such normalisation of a training dataset, if we wish to test an independent test sample REV from a given tissue and gene we will have to normalise the REV using the offsets and scalings that were used in the timecourse normalisation of the training data for this tissue and gene. Such normalisation of test data is called *timecourse-matched*.

There is, however, a cost in using timecourse-matched normalisation because the test data from a particular tissue has to be normalised using the adjustments calculated for that tissue in the training data. This means that one can only use test data for tissues where we have a training time-series. Moreover, when using timecourse-matched normalisation on test data it is crucial that the training data are produced by the same transcriptomics platform.

Intergene normalisation avoids this. When timecourse normalisation is unnecessary or impossible because we do not have a training data set for the test data tissue, the data is normalised using *intergene normalisation* where, if *g* = (*g*_*i*_) is a REV, the normalised levels are given by ${\hat{g}}_{i}=\left(g_{i}-\mu \right)/\sigma$ where *μ* and *σ*^2^ are the mean and variance of the entries *g*_*i*_. Essentially, this maps the REV onto its shape as a vector. It is also possible to usefully combine timecourse and intergene normalisation (see Table 1). Though the use of timecourse normalisation typically improves timing performance, we will show that intergene normalisation can also be remarkably effective (e.g., see Table 1).

**Table 1:** Mean and median absolute timing errors for the training datasets. Column 1 shows the normalisation used. Columns 2 and 3 show respectively the mean and median absolute timing error. Columns 5 and 6 show the mean and median absolute timing error after a correction is made using the timing displacement for the tissues or individuals as relevant. **A-E**. The apparent timing errors for the training datasets when a leave-one-out cross-validation approach was used. For the Zhang *et al*. microarray data [1] we compare using all the data (2h resolution) to only a subset giving 6h resolution. **F**. Timing results for Zhang *et al*. RNA-seq test data [1] when Zhang *et al*. microarray [1] is used as training data. **G**. As **F**. but with datasets swapped.

**A. Zhang *et al*. 2014 Microarray, mouse** [1]				
normalisation	mean	median	corr. mean	corr. median
intergene	1.39h	0.93h	1.35h	0.94h
timecourse (2h)	0.89h	0.70h	0.77h	0.52h
timecourse (6h)	0.78h	0.63h	0.61h	0.53h
both	0.90h	0.60h	0.84h	0.59h
**B. Zhang *et al*. 2014 RNA-seq mouse** [1]				
intergene	1.60h	0.80h	1.51h	0.82h
timecourse	0.68h	0.46h	0.58h	0.59h
both	0.64h	0.27h	0.66h	0.56h
**C. Bjarnason *et al*. human** ([30] **and Note A in** S1 Appendix)				
intergene	1.52h	0.73h	0.95h	0.75h
timecourse	1.14h	0.86h	0.70h	0.62h
both	1.06h	0.48h	0.67h	0.48h
**D. Mure *et al***. [2] **trained on central 18 tissues, baboon**				
intergene	2.43h	1.87h	2.27h	1.51h
timecourse	1.49h	1.11h	1.21h	0.90h
both	1.49h	0.90h	1.23h	0.86h
**E. Mure *et al*. trained on all 33 tissues, baboon** [2]				
intergene	2.53h	1.84h	2.36h	1.65h
timecourse	1.53h	1.13h	1.24h	0.88h
both	1.53h	0.94h	1.27h	0.92h
**F. Test: Zhang *et al*. RNA-seq** [1]. **Training: Zhang *et al*. microarray** [1]				
timecourse	0.83h	0.51h	–	–
**G. Test: Zhang *et al*. microarray** [1]. **Training: Zhang *et al*. RNA-seq** [1]				
timecourse	0.86h	0.67h	–	–

We can also apply such timecourse normalisation to test data when this contains a time series; as several experimental model datasets do. However, any difference in amplitude between the training and test dataseries is then removed. On the other hand analysis using timecourse-matched normalisation for the test data maintains such a change in amplitude.

**Fig 1:**
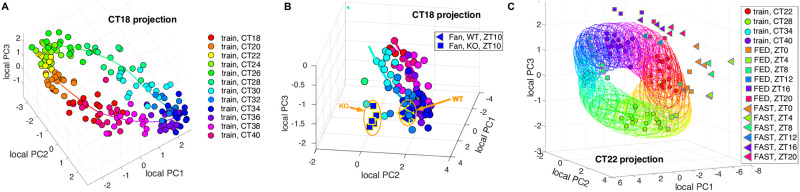
The color of data points etc (when not black) corresponds to the time when the data was sampled. This coloring is used in a consistent way across all figures. **A-C**. Using local PCA projection to visualise data. The identity of each data point can be read from the legends to the right of each example. Only one projection for each example is shown but the differently timed projections have a similar quality. Examples showing all of the projections are in Figs E & G in S1 Appendix. **A**. The CT18 local PCs of the Zhang *et al*. microarray data [1] but using timecourse normalisation. **B**. A detail from a projection of the the Fang *et al*. test data [32] together with the Zhang *et al*. training data [1] as in A showing coherence of the Fang *et al*. WT data [32] and the gap between this and the KO data. Intergene normalisation is used. **C**. The Kinouchi *et al*. RNA-seq skeletal muscle test data for FED and FAST mice [34] plotted against the Zhang *et al*. RNA-seq training data [1]. Timecourse and timecourse matched normalisation is used. The ellipsoids shown are of the form (*x* − *μ*)^*T*^Σ^−1^(*x* − *μ*) = *ε* where *μ* is the mean of the estimated *P*(*g*|*t*) and Σ is its estimated covariance with *ε* chosen so that the ellipsoid should contain 97.3% of the training data (i.e. 3 standard deviations). This enables visualisation of the variation and covariation in the data.

**Fig 2:**
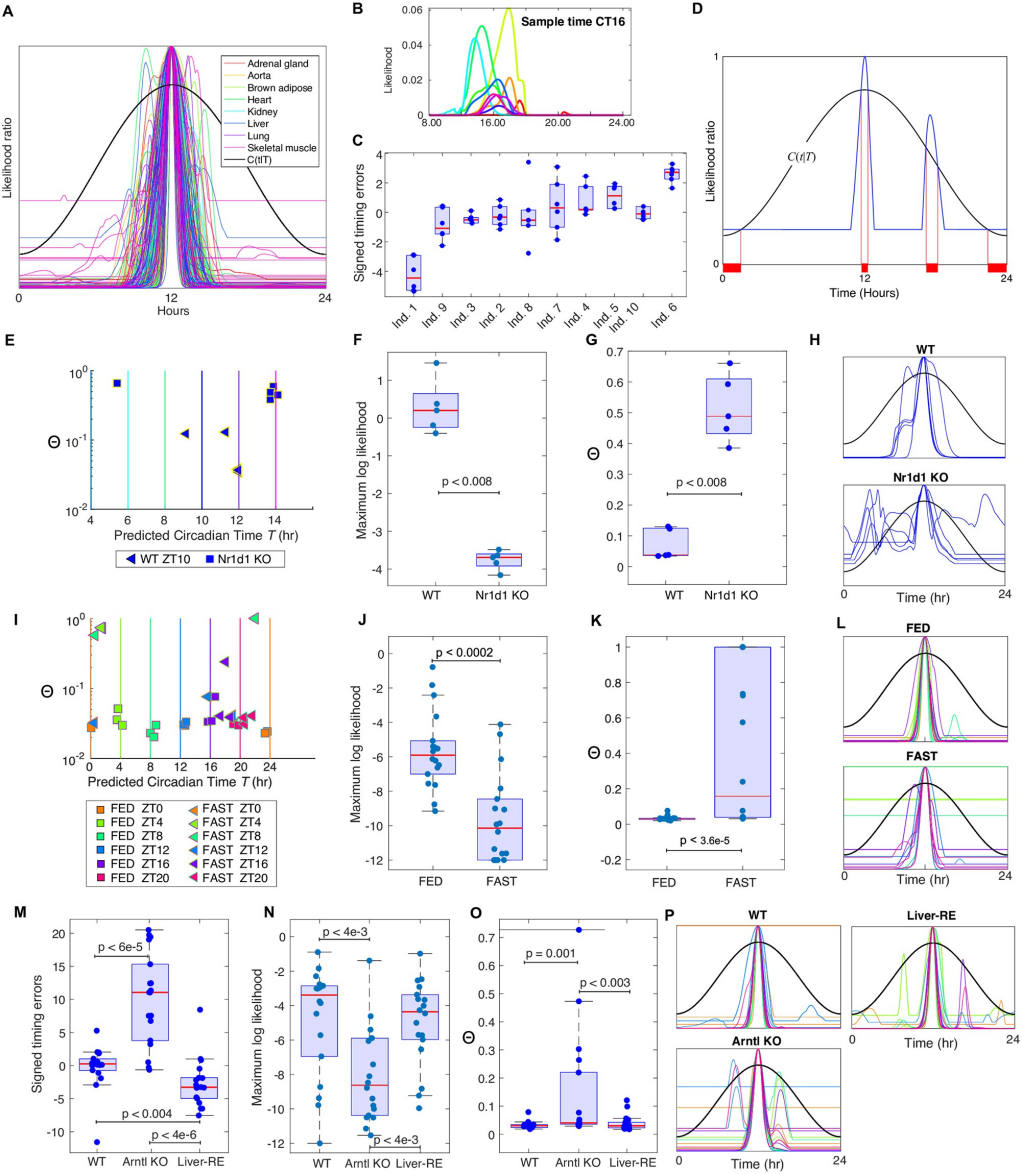
**A**. The centred LRFs for the Zhang *et al*. mouse microarray data [1] using a leave-one-out analysis. They are centred in that the maximum of the curve is moved to noon. This makes the shapes of the curves clearer and more comparable. The black curve is the curve *C*(*t*|*T*) for *T* = 12 (Methods) that is used in the calculation of Θ. **B,C**. **A Leave-one-out analysis of the Bjarnason *et al*. oral mucosa data** [30]. **B**. Examples of the likelihood curves. **C**. Boxplots showing the apparent timing errors found for each individual ordered by their means. This shows the substantial timing displacements of some individuals. **D**. Showing how Θ is calculated using the LRF and the curve *C*(*t*|*T*). Θ is the proportion of time the LRF spends above *C*(*t*|*T*) i.e. the proportion of the time in the horizontal red curves. This is contributed to by the LRF around the highest peak, and by any secondary peaks or flat regions that go above *C*(*t*|*T*). **E-H**. **Analysis of the Fang *et al*. *Rev-erb-α* KO data** [32]. Uses *l*_thresh_ = −5. **E**. Plots of the Θ value against the estimated time *T*. The vertical lines show the true time with colours indicating the sampling time. WT timings are close to the true sample times and the KO times deviate from them. **F and G**. Boxplots of the maximum likelihood and Θ values showing significant differences between the WT and KO groups with *p*-values from the Wilcoxon rank sum test calculated using the Matlab ranksum function. Note that the smallest MLs are around *e*^−4^ which is why *l*_thresh_ was taken to be -5. Taking *l*_thresh_ = −4 gives entirely similar results. **H**. The centred LRFs for the WT and KO samples. **I-L Analysis of the Kinouchi *et al*. FED/FAST skeletal muscle data** [34]. This analysis used a logthresh of −12. The plots J-L are as for F-H but for the Kinouchi *et al*. data [34]. **M-P Analysis of the Koronowski *et al*. data** [39]. comparing WT, Arntl KO and Liver-RE data using *l*_thresh_ = −12. **M** The signed error boxplots show the timing dysfunction in the KO data as well as good recovery in the reconstituted Liver-RE clock but with a clear phase advance. **N,O,P** Boxplots of ML and Θ values, and centred LRFs for the three genotypes.

**Fig 3:**
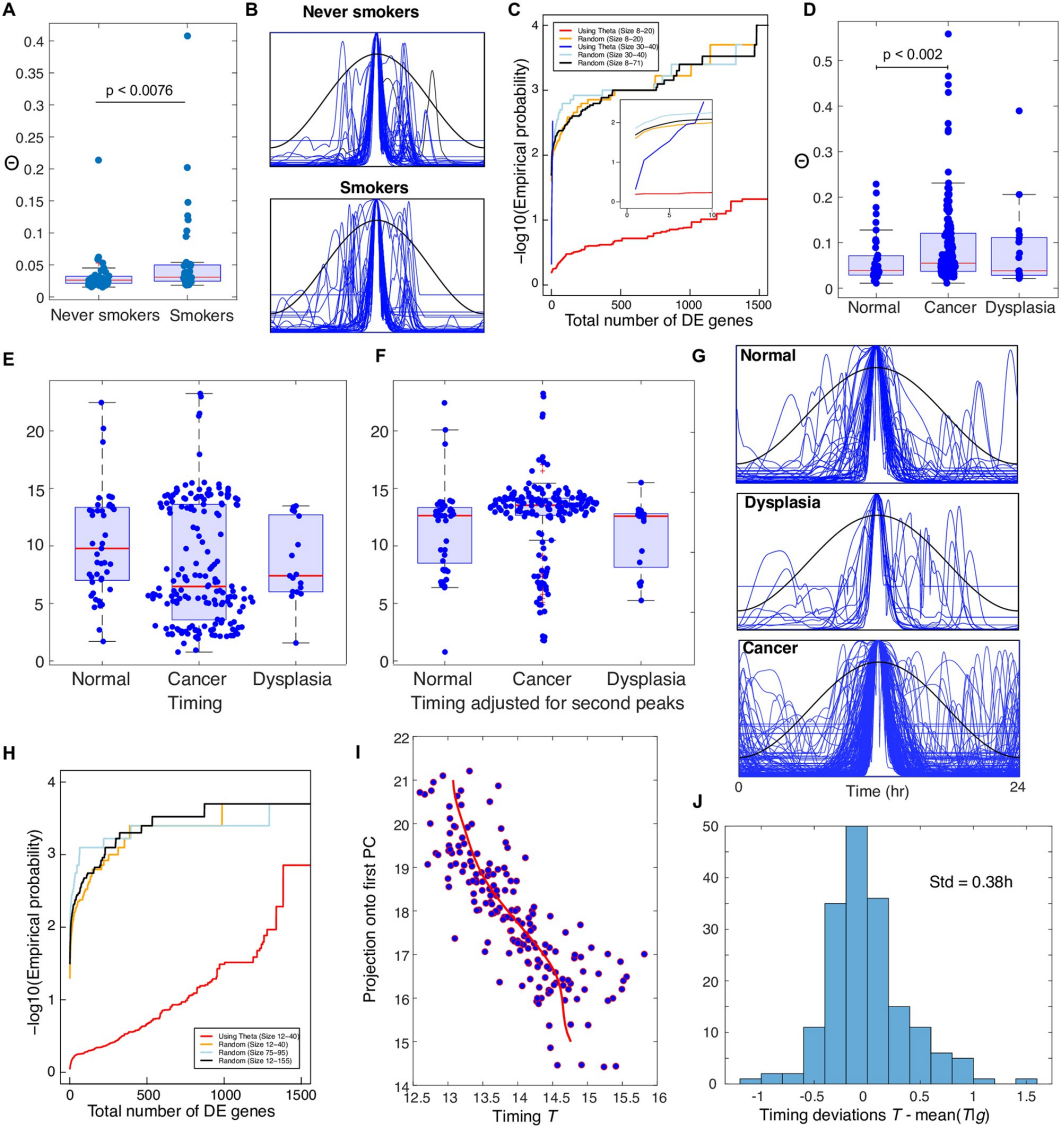
**A-C**. **Analysis of the Boyle *et al*. data** [43]. **A**. Boxplots of the Θ values for the smoker and never smoker individuals showing a statistically significant difference in the distributions. There is no statistically significant (Wilcoxon test) difference for the maximum likelihoods (Fig L(B) in S1 Appendix). **B**. The centred likelihood curves for the smokers and never smokers. **C**. The black, orange and light blue curves are estimates of the probability *p*_rand_ for random choices of the bad clock group of different sizes as in the legend. The red curve is for *p*_Θ_(*m*). There were 5000 iterations for each curve shown. which gave a similar result to 10,000. The number of DE genes was decided using the BH adjustment method with p < 0.05 without any restriction on the minimum log fold change. The inset shows a blow up of these curves for *m* ≤ 10. From the blue curve (*p*_Θ_(*m*) for 30 ≤ *m* ≤ 40) we see that for this range of *m* (unlike 8 ≤ *m* ≤ 20) it is very likely that only a very small number of DEGs are found. For the 66% of cases where a DEG is found there is a 99% chance that *PER3* is among them and a 68% chance of *NR1D2* being present. **D-I**. **Analysis of the Feng *et al*. data** [50]. **D**. Boxplots of the Θ values for the samples from individuals in the normal, cancer and dysplasia subgroups. These show a statistically significant (Wilcoxon test) difference in the distributions between the normal and cancer groups and the cancer and combined normal and dysplasia subgroups. **E**. Boxplots showing the predicted timing of the samples. **F**. Boxplots showing the predicted timing when all samples timed as before 7am and with a second peak are given the timing of the second peak. Of the 108 such samples 93 have moved. This suggests that the mistimed samples are primarily so because the wrong peak has a higher likelihood. **G**. Centred LRFs for the three subgroups. **H**. A study of differential effects between between those *n* individuals with the worse clocks according to the Θ stratification and those with better clocks. The black, orange and light blue curves are estimates of the probability *p*_rand_ as in C above but for the Feng *et al*. data [50]. The red curve is for *p*_Θ_(*m*). **I**. Scatter plot of the projection $\tilde{g}$ of each REV in the Feng *et al*. data [50] with 12 < *T* < 16 (after using the second peaks if the first gives *T* < 7) against timing *T*. The red curve is a kernel smoothed estimate of the mean of $P(T|\tilde{g})$. **J**. Distribution of the deviations in H. For each data point this is the horizontal difference between the data point and the red curve. A simple analysis shows that this is largely independent of $\tilde{g}$ and hence its standard deviation can be used as an upper bound for that of *P*(*T*|*g*).

**Fig 4:**
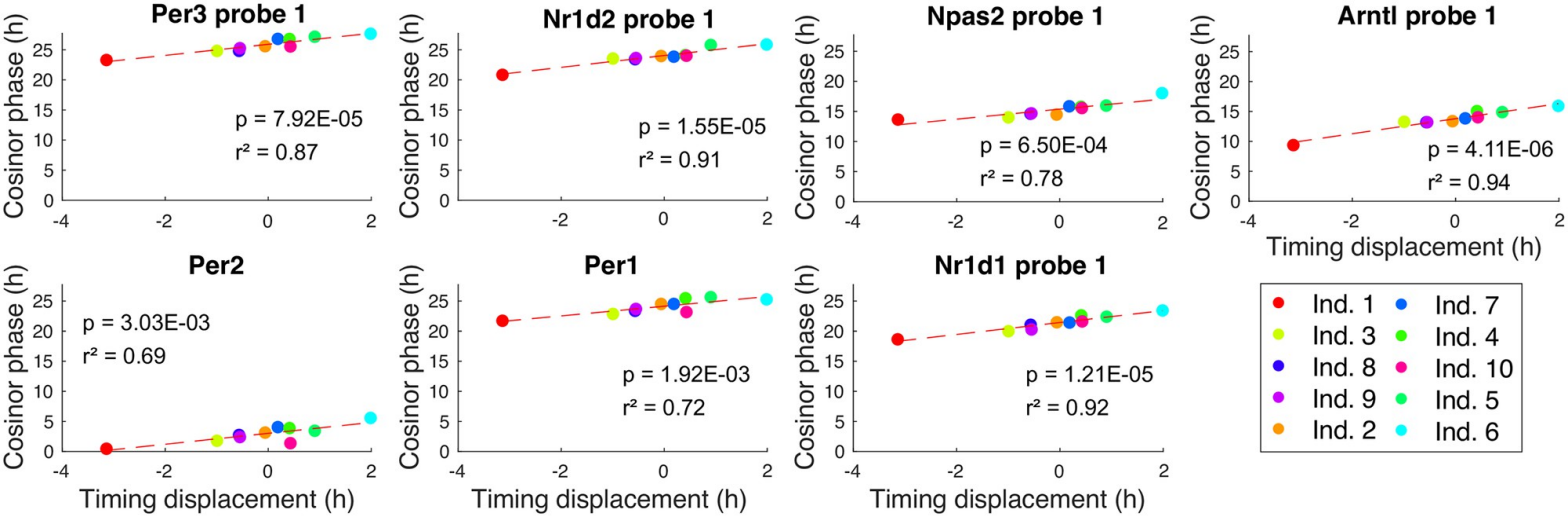
Examples of PCP plots for the Bjarnason *et al*. data [30]. These (full set in Fig M in S1 Appendix) show the strong linear relationship between the REP gene phases and the timing deviations in this data. Each point corresponds to an individual. The regression was carried out using Matlab’s fit function and Cosinor [52] was used to estimate the gene phases from the time series of each individual. The *p*-values test the hypothesis that the slope of the line is non-zero and are given by the F-test using the Matlab functions coefTest and fitlm.

**Fig 5:**
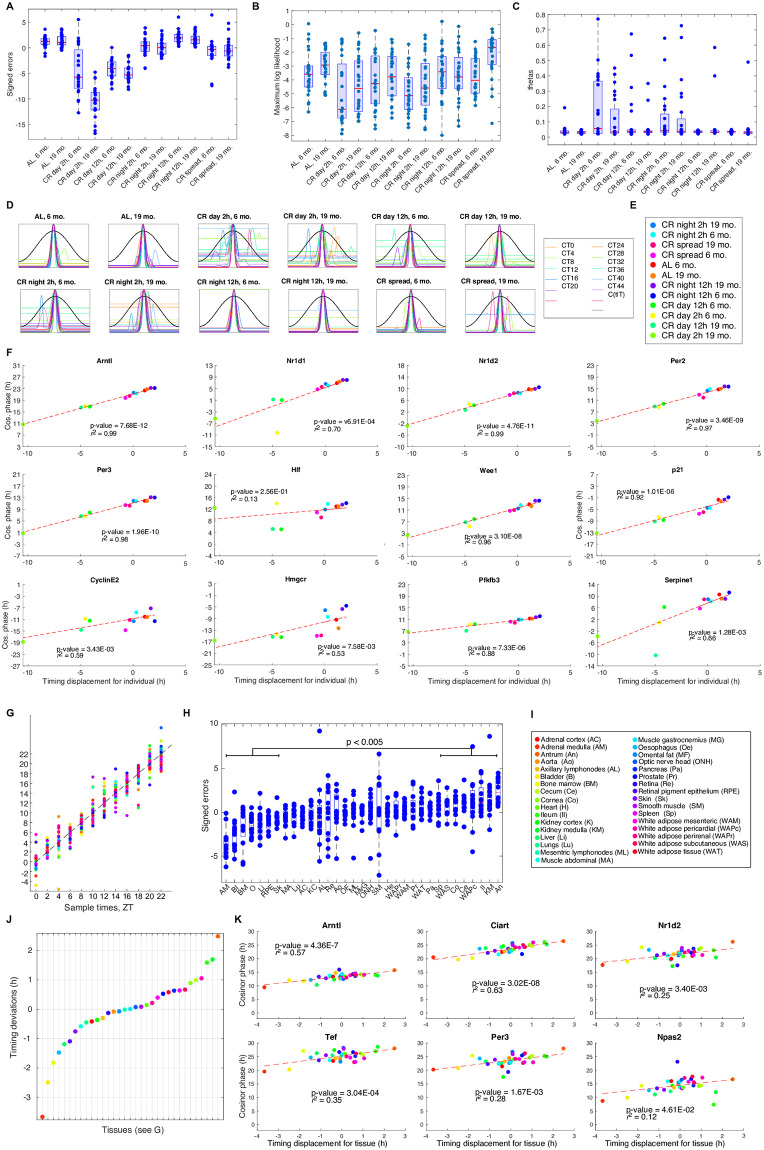
**A-E**. **Analysis of the Acosta-Rodríguez *et al*. data** [37]. This data is analysed as test data using timecourse-matched normalisation and timecourse normalised Zhang *et al*. RNA-seq data [1] for training. We use *l*_thresh_ = −8. The timing, Θ and ML for each sample point shown is a suitably averaged value for the two replicates with the same feeding condition and age. **A**. Box plots of the apparent timing error for the different conditions and ages. The mean value of each box plot gives the timing displacement for the condition and age. Only 12 of the 66 comparisions are not significant at the *p* = 0.05 level using the Wilcoxon test. **B**. Box plots of the ML for the different conditions and ages. **C**. Box plots of the Θ value for each condition and age. See Table D in S1 Appendix for statistical analysis of the differences. **D**. Centred LRFs for each condition and age. **E**. Legend. **F**. PDP plots for the genes in the REP, some cell cycle genes and some of the genes highlighted in [37]. See Fig N in S1 Appendix for more information. The gene phases were measured by Cosinor [52]. **G-K**. **Analysis of the Mure *et al*. data** [2]. In each plot the color corresponds to the tissue as shown in I. The data from the central tissues is used for training. **G**. TimeTeller predicted time *T* vs the sample time using leave-one-out analysis for each sample from the 33 tissues. **H**. Box plots of the signed apparent errors for the samples for each of the 33 tissues in order of increasing timing displacement. Each of the 7 leftmost boxplots is significantly different from each of the 7 rightmost boxplots at the *p* = 0.005 level. **I**. Legend for G-K. **J**. Timing displacements for each tissue. **K**. Some examples showing PDP plots of the gene phases and the TimeTeller timing displacement for all 33 tissues from Mure *et al*. [2]. The *p*-value and *r*^2^ values for the other genes are in Table C in S1 Appendix. Note that the *r*^2^s (Fig 4) for core clock genes are much smaller than those for the Bjarnason *et al*. human data [30] and the Acosta-Rodríguez *et al*. data [37] in F.

Similar considerations to the above apply when combining data across individuals instead of tissues as we do with the Bjarnason *et al*. human data ([30] and Note A in S1 Appendix). Timecourse normalisation can also be very useful when analysing microarray data and we have found it necessary when the training and test data come from different microarray platforms (e.g., as in Figs C and D in S1 Appendix). Table A in S1 Appendix. summarises the normalisations that were used for all the analyses shown in the Figs 1–5.

#### Estimating the clock statistical structure of the training data

We mentioned above that the joint distribution *P*(*t*, *g*) of time *t* and clock gene state *g* or some representative of it characterises the statistical structure of a clock. This distribution is always associated with the training data and test data is analysed using it. In fact, rather than *P*(*t*, *g*) we will mainly be interested in the two conditional distributions *P*(*g*|*t*) and *P*(*t*|*g*) associated with it. TimeTeller aims to use the training data to estimate *P*(*g*|*t*) for all times *t* across the day as explained in Methods, and Fig E and Note C in S1 Appendix. Moreover, *P*(*g*|*t*) and *P*(*t*|*g*) are related by Bayes’ law (Note D in S1 Appendix) and in the case of clocks this boils down to the fact (since times *t* are equally probable) that, as functions of time *t*, *P*(*t*|*g*) is approximately proportional to *P*(*g*|*t*). Therefore, for any clock gene state *g* from training or test data, we can use knowledge of *P*(*g*|*t*) to determine the temporal shape of *P*(*t*|*g*). Furthermore, as we explain in Note D in S1 Appendix, the variance of *P*(*t*|*g*) depends crucially on the covariance structure of the clock genes, i.e. the covariance matrix of *P*(*g*|*t*). Our tool is constructed to use this understanding. Finally, we note that the stochastic dynamics of the system around its periodic attractor modified by measurement noise sets the nontrivial structure of this covariance matrix. From theoretical considerations [31], if the measurement noise is not too large, we can expect that the covariance matrix has rapidly decaying eigenvalues, an observation that will justify our dimension reduction from *G* to less dimensions that is discussed below (also see Fig F in S1 Appendix).

### Multidimensional visualisation provides important information about phenotype

When constructing the probability model, the TimeTeller algorithm projects the G-dimension REVs into fewer dimensions using a local version of principal component analysis (Methods, and Note E and Figs E and F in S1 Appendix). This gives a different projection for each time in the dataset and the algorithm extends this to all times around the day. If for the *G*-dimensional data the distributions *P*(*g*|*t*) are approximately multivariate normal (MVN) then the corresponding distributions of the projected data optimise the capture of the dominant gene-gene correlations after projection (see Methods). We find that for our datasets *d* = 3 is sufficient for this (e.g., see Fig F in S1 Appendix) and the resulting 3-dimensional model of the clock provides a very informative visualisation.

Fig 1A shows such a visualisation for the mouse multi-organ microarray training data from Zhang *et al*. [1] when timecourse normalisation has been applied. TimeTeller actually produces such a local projection visualisation for each time in the training dataset as shown in Figs E and G in S1 Appendix but normally inspection of just one of these is adequate and we only show one in Fig 1. With each such visualisation we also show the curve given by the means of the estimated distributions *P*(*g*|*t*) as *t* varies over the day. Also in such plots we often provide for a sample of times *t* an ellipsoid showing the covariance structure of the estimated distribution *P*(*g*|*t*) (see caption of Fig 1). We color the training data points and mean curve by time with a color coding as given in the legend of Fig 1 using the sample time for the data points. The same color coding is used throughout the paper.

Fig 1B plots microarray test data from Fang *et al*. [32] comparing it with the Zhang *et al*. microarray training data [1] in Fig 1A. This test data compares liver samples of *Nr1d1* (*Rev-erb* *α*) knock-out (KO) and wild-type (WT) mice entrained to light-dark (LD)12:12 cycles. The gene *Nr1d1* is a core clock gene of the mammalian circadian clock important in one of the interlocked feedback loops and a key link to metabolism [33]. Knocking it out leaves a functional but perturbed clock when compared to WT mice [32]. Since *Nr1d1* is a member of the default REP it would not be surprising that TimeTeller could distinguish *Nr1d1* KO mice from WT mice, and indeed this is the case. Therefore, for this validation, we exclude *Nr1d1* from the REP genes. The visualisation shows that while the WT data appears to fit well with the training data, the KO data has a consistent substantial difference. TimeTeller is able to detect this apparent difference in each of the four KO samples and shows a coherent difference from WT. This suggests that the *Nr1d1* KO mice have a significantly perturbed clock when compared to WT mice (Fig 1B). However, it is still somewhat functional as it gives approximately correct timing and the level of sample variation between WT and KO is similar. We investigate this further below.

The other test data we visualise (Fig 1C) in this figure is from Kinouchi *et al*. [34]. This contains samples analysed by RNA-seq from mouse skeletal muscle taken around the clock in LD 12:12 [34], and compares mice that had been fed *ad libitum* (FED) with mice that had been starved for exactly 24 hrs prior to point of sampling (FAST). On the one hand, while FED samples align with the RNA-seq training data, the FAST samples are substantially perturbed (Fig 1E). On the other hand, the FAST samples show consistency in that for a given sample time they tend to cluster together. A similar visualisation for the liver samples from [34] is given in Fig H in S1 Appendix. It should be noted that the test samples from Kinouchi *et al*. [34] have been collected in LD whereas the training dataset was collected on the first three days in constant conditions. Interestingly, there is little difference between FED (control) and training dataset mice, which might be due to the fact that the free-running period of these WT C57Bl/6 mice is around 23.8 hours.

Other examples demonstrating the utility of such visualisation are discussed below.

### Analysis of single test samples

TimeTeller’s estimate of *P*(*t*|*g*) from the training data is used to analyse test data. For a normalised test data REV *g* our estimate *L*_*g*_(*t*) of *P*(*t*|*g*), which we regard as a function of *t*, is referred to as the *likelihood curve* (LC) for the corresponding transcriptomics sample. The quantities for functionality assessment are associated with this LC. For example, we define the *internal phase* *T* of a test REV *g* as the time at which the estimated likelihood function *L*_*g*_(*t*) ≈ *P*(*t*|*g*) is maximal i.e., the maximum likelihood estimate. Given *T*, we define the *likelihood ratio function* (LRF) as *R*_*g*_(*t*) = *L*_*g*_(*t*)/*L*_*g*_(*T*), i.e. it is the LC but normalised so that the value at the maximum is 1. The internal phase can be compared with the SCT but, as noted above, there may be consistent phenotypic deviations of *T* from the SCT in genetically heterogeneous populations.

It is important to emphasise that when we analyse test data the results for any test data sample are independent of the results for any other test data sample. This is because the calculation of the likelihood curve of a test data sample only involves the probability model and the test data sample and has nothing to do with the other test samples. Therefore, the result for any test sample will be exactly the same as if it were the only sample in the test dataset.

Fig 2A shows the estimated LRFs for the Zhang *et al*. microarray data [1]. Each LRF’s highest peak is centered at 12noon to enable visual comparison of many LRFs, a plotting technique used throughout the figures. Many examples of estimated LCs and LRFs can be seen in Figs 1–5 and the S1 Appendix. The resulting predicted timing plotted against the sample time is shown in Fig 2A together with the times corrected to allow for the chronotype explained in the section below on timing. LCs for the Bjarnason *et al*. human training data [30] are shown in Fig 2C and Fig I in S1 Appendix.

These show the general form of the LCs and demonstrate that one can clearly observe qualitative differences between one individual’s LC and those of the others.

#### Apparent timing errors and timing deviations in the training data

For each training dataset we used an appropriate leave-one-out cross-validation approach to compare the sample collection time *T*_*a*_ with the estimated time *T* and evaluated the apparent timing errors *T* − *T*_*a*_ for each sample. The mean and median absolute timing errors (MAEs) for the training datasets are shown in Table 1.

We then analysed how the mean timing error varies with tissue, individual or condition to see if there is a consistent timing deviation for any of these. When these deviations are clear and statistically significant we call the mean of them the *timing displacement* of the tissue, individual or condition. We show below that for the mouse and human training datasets the observed timing displacement is associated with coherent phase changes in the genes. Therefore, in assessing the performance of TimeTeller the apparent timing errors should be corrected to take account of this. The timing displacements of the different mouse tissue in the Zhang *et al*. data [1] are relatively small (Fig G(G) in S1 Appendix) but, for the more genetically heterogeneous human population of the Bjarnason *et al*. data [30], we found significant and consistent timing displacements on the individual level (Fig 2E and Fig I in S1 Appendix). When the apparent errors are adjusted for this they are often substantially reduced (Fig 2D and Table 1). For this data this reduction is of the order of 50%. Table 1 shows that timecourse and timecourse then intergene (both) normalisations are performing significantly better than intergene alone.

It is difficult to compare performance with that of the published algorithms mentioned above as they have been used on different datasets collected under different conditions and there has been relatively little work on time-stamped genetically heterogeneous data. The Zhang *et al*. microarray dataset [1] was also analysed by ZeitZeiger and the mean absolute errors on cross-validation were between 0.6h and 1.1h [15]. On these tissues the results for timecourse normalisation with TimeTeller are very similar to those of ZeitZeiger (Table B in S1 Appendix). Moreover, TimeTeller’s apparent timing errors for the genetically heterogeneous human data compare well with those found in other studies which typically have a median absolute error (MdAE) greater than 1.4h. For example, in the study [23] the 1-sample method had a MdAE of 1.6h and the 2-sample method had a MdAE of 1.4h-1.7h and when CYCLOPS was validated against pre-frontal cortex biopsies with annotated time in [18] the MdAE was 1.69h. In an impressive application to data from four distinct human studies TimeSignature [19] reported MdAEs between 1.21h and 1.49h although this requires two samples for each individual. While TimeTeller’s timecourse normalised results for the genetically heterogeneous Bjarnason *et al*. [30] and Mure *et al*. data [2] (Table 1) compare favourably with these results we do not wish to claim timing superiority as there is great heterogeneity in the studies giving rise to the data that was analysed and in the transcriptomics platforms employed.

#### Maximum likelihood ML

Given a test sample REV *g*, the value of ML = *L*_*g*_(*T*) (i.e. the maximum likelihood of *g*) is a key diagnostic as, if *M* denotes the maximum value of the distribution *P*(⋅|*T*), we can regard λ = log(ML/*M*) as a likelihood ratio test statistic for a pure significance test of the hypothesis that *g* is drawn from the training clock. Thus, a low value of ML relative to the values obtained by training or control data is indicative of the fact that *g* comes from a clock that is substantially different. We refer to dysfunction of this kind as *low ML* (lowML). An initial evaluations of the ML values for both training and test data is a key first step of an analysis using TimeTeller.

#### Dysfunction metric Θ

Statistical theory tells us how to estimate the confidence interval for the maximum likelihood estimator *T* of internal timing for any given degree of confidence using the LRF (Methods and Note D in S1 Appendix). The variance of *T* arises because *g* is a random sample from the clock at time *t* and we want to know how *T* will vary with other such samples because high variance implies imprecise timing. We call such dysfunction *high variance timing* (highTvar). The Cramér-Rao Theorem [35] gives a lower bound for variance in terms that can be related to the LRF (Note D in S1 Appendix). Our metric Θ is the proportion of time in the day that the LRF spends above the curve *C*(*t*|*T*) defined in Methods and is associated with the length of such a confidence interval (Note D in S1 Appendix) and therefore Θ gives an assessment of this sort of dysfunction and higher Θ is associated with higher dysfunction.

However, our likelihood curves often contain structures that are relevant to assessing dysfunction but which are not covered by this aspect of statistical theory. One important case is where when *g* has significant dysfunction of type lowML and the other is where the LC and LRF contain significant secondary peaks that have a lower likelihood than that at *T*. Complex data sets from diseases such as cancer can contain all of these dysfunction signatures with some samples displaying a single type and others a mixture of more than one. As well as seeking to characterise the type of dysfunction, we attempt to construct a statistic that integrates the different types into a single measure. This is our dysfunction metric Θ. As defined by us (see Methods) this metric will contain a contribution from all of these aspects that are present and therefore *ML* and Θ are not independent. We discuss this further in the following sections after discussing the values of Θ and ML in training data.

#### Θ and ML for training data

To continue the evaluation of TimeTeller’s LCs, and the corresponding dysfunction metrics Θ and ML we first tested it on the Zhang *et al*. [1] and Bjarnason *et al*. [30] training datasets using the appropriate leave-one-out cross-validation approach. The results showed consistently low Θ values and relatively high maximum likelihoods across tissues for the genetically homogeneous mouse datasets and genetically inhomogeneous individuals for the human data (Figs G, I and J in S1 Appendix).

### Multiple tools for assessing functionality in test data

Importantly, this consistency of good apparent timing errors, high ML and low Θ was also observed in the analysis of the various WT/control components of the test data sets considered. For example, using the Zhang *et al*. microarray data [1] for training and intergene normalisation our analysis of the microarray timecourse control dataset created by LeMartelot *et al*. ([36] and Note A in S1 Appendix) produced a mean absolute error for time estimation of less than one hour and Θ values similar to those found in the training data. Similar results were found for the Acosta-Rodríguez *et al*. data [37] for *ad libitum* fed mice using the Zhang *et al*. RNA-seq data [1] for training and timecourse-matched normalisation, and for liver microarray test data from Hughes *et al*. [38] after training with the Zhang *et al*. microarray data [1] (Fig C in S1 Appendix). For the latter we used timecourse normalisation for the training and test data as the microarray platforms are different, demonstrating good results across different platforms.

To further test the use of TimeTeller across different transcriptomics platforms we carried out a cross-validation experiment where we trained TimeTeller on the Zhang *et al*. microarray data [1] and used this to test the Zhang *et al*. RNA-seq data [1] and vice-versa (Fig 1A and Fig D in S1 Appendix). This not only tests the robustness of our approach but also examines the effectiveness of timecourse normalisation in allowing us to work across different transcriptomics technologies. The timing results are given in lines F and G in Table 1 with small mean and median errors of a size compatible with the within-dataset leave-one-out analysis. As well as the relatively small timing errors we observe informative visualisation and consistent Θ values.

Across the various datasets we consider, this good timing, high ML and low Θ for WT/control test data almost always differed from that found for the perturbed test data. For example, in Fig 2H–2O we consider the timing, ML and Θ diagnostics for the Fang *et al*. [32] and Kinouchi *et al*. test data [34] discussed above and use them to illustrate how to gain more insight into dysfunction. In such an analysis one should start with an assessment of the MLs and an inspection of the LRFs for training, control and test data.

From this one can choose an initial value for the important parameter *l*_thresh_ using the approach described in Methods and Note C in S1 Appendix. This parameter truncates the likelihood curves so they do not go below exp(*l*_thresh_) and this plays an important role in ensuring that the incorporation of the local likelihoods into a global one (see Methods) is not wrecked by inaccurate and uninformative exceptionally low local likelihoods.

For the Fang *et al*. data [32] and the Kinouchi *et al*. skeletal muscle test data [34] we see that the ML values for the perturbed test data are significantly lower than those for the control test data (Fig 2J and 2N) suggesting that dysfunction of the lowML type is present in the perturbed systems. For the test data from Fang *et al*. [32] we also observe significant differences between the WT and *Nr1d1*KO samples for the timing and Θ diagnostics (Fig 2H–2K). The timing *T* of the KO observations is significantly further from the true timing (Fig 2H). Analysis as in Methods and Note C in S1 Appendix suggests setting *l*_thresh_ around -5 but the results are very similar for any value between -4 and -7. However, the centred LRCs also indicate that there is a significant amount of highTvar dysfunction in the KO sample because of the second peaks and increased width of the LRCs near the maximum.

For the Kinouchi *et al*. skeletal muscle data [34] the FED samples show uniformly small errors in timing *T* (MAE 0.44h) and uniformly low Θ values (Fig 2L and 2M). In contrast, the timings *T* of the FAST samples are clustered around ZT 18–24 reflecting the tight clustering seen in the visualisation (Fig 1E) and the MAE is significantly greater at 4.04h. So far as dysfunction is concerned, the situation is somewhat different from the Fang *et al*. data [32] since, although there are also significant differences in ML and Θ values between FED and FAST (Fig 2I–2L), there are no second peaks in the centred LRFs contributing to Θ (Fig 2L). Consequently, the primary difference between the FED and FAST samples is due to the significant difference in the MLs. Thus, only substantial lowML type dysfunction is present. The stratification by Θ in Fig 2K reflects this.

The Kinouchi *et al*. data [34] provides a very informative example of how the choice of the parameter *l*_thresh_ works because the maximum likelihoods ML for both the FED and FAST liver data are significantly higher than that for the skeletal muscle data discussed above and this means that different values of *l*_thresh_ are appropriate. The discussion in Note C and Fig H in S1 Appendix shows that the value for the liver data should be substantially larger at -6 or -7 rather than -12. When this value is chosen the results for the Kinouchi *et al*. liver data [34] are similar to those above for the skeletal muscle data (Fig 2I–2L).

In order to understand the effect of fasting on the amplitude of core clock components Kinouchi *et al*. [34] needed to treat the data as though the FAST samples belonged to a continuous time series even though each timepoint was proceeded by 24 hours of starvation. This underlines a significant extra advantage of TimeTeller because the FAST test samples can be considered independently from one another.

Koronowski *et al*. [39] compared the liver transcriptomes of wild-type (WT) with whole body *Arntl* deficient mice (KO) or *Arntl* KO mice with liver-specific *Arntl* reconstitution (Liver-RE). Their data enables us to test TimeTeller’s sensitivity to not only the substantial KO perturbation but also the much subtler one of the Liver-RE. Our analysis shows statistically significant differences in timing between WT, KO and Liver-RE including the phase advancement noted in [39] of the Liver-RE clock relative to WT (Fig 2M–2P). The MLs (resp. Θs) for the KO data are significantly smaller (resp. larger) than for both the WT and Liver-RE data with no significant difference between WT and Liver-RE (Fig 2N and 2O). However, the LRFs (Fig 2P) suggest a clear difference between WT and Liver-RE data in that, unlike WT, about half of the Liver-RE samples have a significant extra peak suggesting a contribution of highTvar type disruption and a hypothesis that this is causing the observed timing change in the Liver-RE data. Significant second peaks are also observed in about a half of the the KO data suggesting a combination of some highTvar dysfunction combined with the significant lowML dysfunction.

#### Analysing stopped clocks

In the Fig K in S1 Appendix we discuss an analysis of two studies (Weger *et al*. [40] and Yeung *et al*. [41]) where the clock is disrupted by either *Arntl* (*Bmal1*) or *Cr1/Cry2* deletion. As well as confirming the observation in Hughey *et al*. [15] that the resulting data show clustering to a narrow range of apparent times for the KO samples, the TimeTeller Θ and maximum likelihood values provide quantitative evidence about the dysfunction caused. This is similar for the two *Arntl* KO datasets but different to that of the *Cry1/Cry2* KO dataset. The two *Arntl* datasets the KO samples have significantly reduced ML values and significantly increased Θ values and inspection of the centred LRFs show that almost all the contribution to Θ in the KO samples comes from flat regions in the LRFs. It follows that the dysfunction is primarily of lowML type with the KO data having moved away from the training clock in a way that gives consistently wrong times. In contrast, the *Cry1/Cry2* KO samples though having similar radically wrong timing, had similar high ML and low Θ values to the control data (Fig K(I,L,O) in S1 Appendix). This confirms the visualisation showing that the KO data sits remarkably close to the mean trajectory of the training clock in a way that indicates that its dysfunction is just in the timing. We therefore hypothesise that the *Cry1/Cry2* KO clock is “frozen” in a particular state very close to a wild-type clock state because it has undergone a SNIC bifurcation (see below). This is an extreme example of where there is significant timing dysfunction where the clock reliably gives the same wrong time but no dysfunction of the lowML or highTvar types. We call this dysfunction type *reliable wrong timing* (relTwrong). We will see other examples of this below where the clock is not stopped.

In a deterministic dynamical system, when a parameter is changed slowly there are only two generic ways that oscillations are killed: the Hopf bifurcation where the amplitude declines to zero, and the saddle-node SNIC bifurcation where, until the bifurcation occurs, the amplitude of the oscillation is maintained but at the bifurcation the system stops at a point on the system’s limit cycle [42]. This insight and the quantification results from TimeTeller suggest our hypothesis that mice deficient in *Cr1/Cry2* have undergone a SNIC bifurcation in the liver clock.

### The potential for the use of the Θ stratification to identify differential effects in patients

It is particularly interesting and important to apply TimeTeller to genetically heterogeneous human data because it allows us to test the idea that it can uncover corresponding heterogeneity in the “clock” phenotype or effects on individuals such as patients.

We firstly consider data from a study of the effects of cigarette smoke on the human oral mucosal transcriptome, In this study (Boyle *et al*. [43]) transcriptomes from buccal biopsies of 39 current smokers (≥ 15 pack-year exposure) and 40 age- and sex-matched never smokers (< 100 cigarettes per lifetime) were analysed and compared. The authors found that smoking altered the expression of numerous genes but none of those found were core clock genes nor did they consider the effect of smoking on the circadian clock. They found smokers had increased expression of genes involved in xenobiotic metabolism, oxidant stress, eicosanoid synthesis, nicotine signalling and cell adhesion and decreases were observed in the genes *CCL18*, *SOX9*, *IGF2BP3* and *LEPR*. It has been reported elsewhere that smoking has an impact on multiple sleep parameters and significantly lowers sleep quality [44–46] and this was confirmed in an experimental study which also correlates poor sleep to inflammation [47] while inflammation has been linked to clock disruption. Moreover, CS exposure has been shown to cause circadian disruption in the lungs of WT mice and this is exaggerated in the *Nr1d1* knockouts [48] and has a connection to *Arntl* [49].

Interestingly, when analysed by TimeTeller (Fig 3A–3C) we see a clear and statistically significant difference between the Θ values of the never smoked and smoking individuals (Fig 3A) which is reflected in the 3D visualisation (Fig L(A) in S1 Appendix). Inspection of the LRFs show that the variations in Θ come mainly from second peaks rather than low ML (Fig 3B). Indeed, the ML values for smokers and non-smokers were not significantly different although the smokers had more observations with a very small ML (Fig L(A-C) in S1 Appendix). The lowest values were around *e*^−11^ suggesting that a *l*_thresh_ of about -12 would be appropriate.

A significant proportion of the smokers had Θ values similar to those of the never-smokers but many had much higher values (Fig 3A). Therefore, we asked if we could identify differentially expressed genes (DEGs) between the individuals with high Θ versus those with lower Θ. To do this we tested for differential gene expression between the *n* worst clocks (defined as the *bad clock group* (BCG)) and the others (*good clock group* (GCG)) adjusting the *p*-value appropriately to allow for the multiple testing. For a fixed *l*_thresh_ in the range from 11 to 13 with *n* between 8 and 20 we found many differentially expressed genes (DEGs) at the appropriately adjusted *p* = 0.05 level including some clock genes (Fig L(D,E) in S1 Appendix). However, the particular genes found were sensitive to changing the value of *l*_thresh_ among the suggested values of -11, -12 or -13 and changing the group size *n*.

We calculated that the probability of finding such numbers of DEGs by chance is extremely low (Fig L(H) in S1 Appendix) and we noticed significant differences between the behaviour when the BCG size was in the range 8 to 20 from that when it was 30 to 40. Therefore, we estimated by simulation the probability *p*_rand_(*m*) of finding *m* or more DEGs by chance when we choose a random group of *n* individuals for our BCG and compared this to the probability *p*_Θ_(*m*) of finding *m* or more DEGs when the stratification by Θ is used to choose the BCG and *l*_thresh_ and *n* are chosen randomly in the ranges -11 to -13 and 8 to 20. We find that uniformly in *m*, *p*_Θ_(*m*)/*p*_rand_(*m*) > 100 (Fig 3C). We get an interestingly different result if we instead let the group size *n* range between 30 and 40. The probability *p*_rand_(*m*) behaves in approximately the same way but *p*_Θ_(*m*) does not (Fig 3C(inset)). For *m* very small *p*_Θ_(*m*) is high but as *m* increases *p*_Θ_(*m*) rapidly decreases to values much smaller than those for *p*_rand_(*m*). There is a 34.26% chance of getting no DEGs but when this is not the case there is a more than 99% chance of getting the gene *PER3* and a 68% chance of getting *NR1D2*. Thus, this analysis identifies two interesting groups of individuals with a nontrivial transcriptional phenotype that distinguishes them from the individuals with good clocks. One of these groups appears to be associated with differential expression of *PER3* and *NR1D2*, genes not identified in the original paper where all non-smokers and smokers were compared.

In conclusion, any link between smoking and clock dysfunction is likely to be complex, but these results suggest that in a genetically heterogeneous population where the effects of a perturbation such as smoking are likely to be diverse, TimeTeller’s Θ stratification can help identify individuals or groups where the smoking effect is significant.

As a final example of this section we consider the distribution of Θ values by disease state for the transcriptomic data of healthy or dysplastic oral mucosa and oral squamous cell carcinoma (OSCC) from Feng *et al*. [50]. Since the lowest ML values were around *e*^−11^ a *l*_thresh_ of -12 was used. The ML values for normals and cancer were not significantly different although the cancer group had more observations with a very small ML (Fig L(F) in S1 Appendix). However, there is a highly significant difference in median Θ values between the cancer group (167 individuals) and the the normal mucosa group (45 individuals) (*p* < 0.002) (Fig 3D). Moreover, there appears to be significant dysfunction in terms of timing estimation (Fig 3E) that can be significantly ameliorated if the second peaks in the LRF is used for timing when the first peak is clearly misleading (Fig 3F, details below). Inspection of the LRCs (Fig 3G) shows that, as for the Boyle *et al*. data [43], the variations in Θ come mainly from second peaks rather than low ML.

As for the Boyle *et al*. data [43] we asked if there are DEGs between the worst clocks in the cancer group (high Θ) and the best clocks within the same group and carried out a similar analysis. For genes in general and BCG sizes *n* between 12 and 40 we find similar results with *p*_Θ_(*m*)/*p*_rand_(*m*) > 100 for the number *m* of DEGs between 2 and 1200 (Fig 3H). Many of these DEGs are associated with gene signatures such as DNA repair, E2F targets, G2M checkpoint and the mitotic spindle. However, we do not find any groups like that for the Boyle *et al*. data [43] (with *n* between 30 and 40) that have very low numbers of specific DEGs.

A study of the estimated timing *T* for this data (Fig 3E) was very informative. The estimates for the normal data are generally between 7 am and 3 pm. A large number of cancer samples have unlikely times well outside the normal working day and the median is clearly much too early. Interestingly, it appears that the mistimed samples are primarily so because the likelihood curve has a second peak (Fig 3G) and the peak giving an unreasonable timing estimate is slightly higher than one giving the best estimate. In fact, there are 109 samples whose timing *T* is before 7am and 93 of these have a second peak. if we replace the timing by that given by the second highest peak, the great majority moved to a time firmly in the early afternoon between 12noon and 4pm (Fig 3F). As a result 74% of all samples then fall in this time slot and only 7% remain before 7am. The analysis in the next section indicates that this corrected timing is likely to be the correct time of sampling to within approximately 0.4h.

### TimeTeller’s precision on non time-stamped cancer data

The only method currently utilised to estimate the precision of timing/phase algorithms is to use time-stamped data and compare the algorithm’s predicted times *T* with the SCT time stamps *t*. However, such a measure of precision is problematic when the individuals, tissues or conditions have a nontrivial molecular chronotype as is the case with the human data considered here and cannot be done if the data is not time-stamped. A related test which avoids these problems is instead to determine the variance or standard deviation of the distribution *P*(*T*|*g*) where *T* is the predicted time and *g* is the relevant REV (Note F in S1 Appendix). This addresses the question of how well the estimated timing *T* is determined by the REV *g*. Interestingly, we can calculate this precision measure even in some cases where we have no timing data and where there is dysfunction and the Feng *et al*. data [50] gives a very informative example of this.

To illustrate this we study the 77% (176 samples) of that data for which the estimated timing *T* after adjustment by second peaks is between 12 noon and 4pm (Fig 3F). We ask if within this data we can see coherent timing structure or not. We can estimate the required standard deviation by carrying out a principal component (PC) analysis of the expression data (see Note F in S1 Appendix) and plotting the projection of these data onto the first PC against the predicted time *T* (Fig 3I). An upper bound for the standard deviation of *P*(*T*|*g*) can be estimated from this (Note F in S1 Appendix) and we obtain an estimate of less than 0.4 hours. If we consider all the deviations from the mean for the timings *T* (given by the horizontal deviation of the relevant data point from the red curve in Fig 3I) across all of the REVs in this data we obtain the distribution shown in Fig 3J. Remarkably, although the data is not timestamped and has significant dysfunction giving rise to significant second peaks, TimeTeller is able to accurately measure the internal phase *T* of the clock as a function of the REV *g*.

We carried out a similar analysis (see Note F in S1 Appendix) and found similar results but a bigger standard deviation of 0.83h for the large breast cancer dataset analysed by Cadenas *et al*. in [51]. In this case there is no need for adjustment for second peaks as 86% of the data has its predicted time *T* between 10am and 8pm (Note F in S1 Appendix). We believe this approach gives a new simple method to assess timing performance.

### Comparing clocks across individuals, conditions and tissues

Current analyses comparing the circadian clock across individuals, tissues and conditions such as the three studies we consider below proceed by analysing the behaviour of the individual interesting genes separately. Such analyses tend to focus on the level of expression and do not take into account correlations between related genes. We asked whether using TimeTeller such an analysis could be done in a more integrated way treating the clock as a noisy multigene dynamical system (and hence using correlations) and whether such an approach uncovers some aspects that are hard to see when done gene by gene. The key results here are that it enables us to identify coherent differences in timing across individuals, conditions and tissues and that using these we can determine in a quantifiable way if the timing differences come from a more or less coordinated change in gene phases.

#### Using TimeTeller to identify a molecular chronotype

The human training data that we consider (Bjarnason *et al*. [30]) involves genetically heterogeneous individuals and therefore we also asked to what extent in this analysis of time-series data we could differentiate systematic variation of timing in an individual or tissue due, for example, to genetic and/or environmental factors, i.e., a *molecular chronotype*.

We observed above that while the Θ and maximum likelihood values are reasonably consistent across individuals, the apparent timing error was not. For some individuals there were substantial timing displacements arising from intra-individually consistent deviations of the estimated time from the sampling time (Fig 2D). For example, the individuals labelled as 1 and 6 in Fig 2D have substantial statistically significant (*p* < 0.003) timing displacements in opposite directions. To further understand this, we hypothesised that the timing displacement of an individual might be largely a result of well-coordinated phase changes in the core clock genes.

If this is the case there should be a definite relation between TimeTeller’s timing deviations and the phase of the genes. Moreover, since this relationship is local in that the timing displacements are small compared to 24 hours, it is reasonable to suspect that it might be approximately linear. Therefore, we tested for a linear relation between the phase variation of the genes in our panel and timing displacement.

In this analysis, we regressed the timing displacement against the phase of each of the genes in the REP (Fig 4) using Cosinor [52] to measure gene expression phase. For all the probes used we observed an approximately linear relationship between timing displacement and the variation in the gene phase with a positive slope (Fig 4 and Fig M in S1 Appendix). For all genes the non-zero slope is statistically significant and the *r*^2^ value is greater than 0.7, and for many genes it is greater than 0.9. The latter measures the proportion of the variation in the gene phase that is predictable from the TimeTeller displacement using the linear relationship. Thus TimeTeller is able to clearly identify coherent and substantial phase variation in the clock genes for each individual across all genes in the rhythmic expression profile. It identifies a clear “chronotype” for each individual and a quantifiable phase difference. Moreover, the strong coherence between the time estimations and the gene phases are further validation of TimeTeller’s time estimation. These results suggest that if the sample collection time is known, by combining the observation of a Θ suggesting good clock function with an advanced or retarded time prediction, TimeTeller can help identify substantial coherent phase variation in an individual’s clock genes from a single sample.

We will utilise such regression plots in the analyses below where we attempt to characterise the nature of the change in the clock caused by different conditions or in different tissues. We call such plots *phase displacement plots* (PDPs).

#### Timing divergences and clock comparisons for time-restricted feeding in ageing mice

Recently, Acosta-Rodríguez *et al*. [37] studied the synergistic effects of various time-restricted feeding protocols with caloric restriction (CR) on the prolongation of life span in mice, focusing on the liver which is a major metabolic target of the circadian clock. After 6 weeks of baseline *ab libitum* (AL) food access, C57BL/6J male mice were subjected to 30% CR. Mice were fed nine to ten 300mg food pellets containing 9.72 to 10.8 kcal every 24 h starting at the beginning of the day (CR-day-2h) or night (CR-night-2h) constrained to consume their food within 2h.

Two additional CR groups were fed a single 300mg pellet delivered every 90 min to distribute the food intake over a 12-h window either during the day (CR-day-12h) or during the night (CR-night-12h). A fifth CR group was fed a single 300mg pellet every 160 min continuously spread out over 24 h (CR-spread). Liver gene expression was profiled using RNA-seq in all six feeding conditions at 6 and 19 months of age. Livers were collected in constant darkness at 12 time points every 4 hours for 48 hours across two circadian cycles. We treat the data from time *t* and *t* + 24 as replicates of a 24h cycle.

Together with a young and old group where feeding was *ad libitum* (AL) this results in 12 feeding conditions. We used TimeTeller to analyse this data asking if it could identify the nature of systemic changes in the core clock between the different feeding×age conditions. We used the Zhang *et al*. RNA-seq data [1] as training data. Thus, all feeding conditions of [37] are regarded as test data. We analysed this using both time-course and timecourse-matched normalisation for the test data. The results are very similar and we give the timecourse-matched results here.

Visualisation showed that the test data fell nicely within the trained distribution close to the mean cycle. Analysis as in Note C in S1 Appendix points to using a *l*_thresh_ of -8. The results on the predicted times *T* showed a substantial timing displacement (Fig 5A) for eight of the conditions with CR-day-2h being the most extreme. Only 12 of the possible 66 comparisons have *p* ≥ 0.05. Moreover, there is a striking apparent age-related difference for the CR-day-2h feeding conditions in that the timing displacements of the 6 month and 19 month mice differ by over 4 hours (*p* < 0.0001).

There are some statistically significant differences between the Θ and ML values found for the different conditions (Fig 5B and 5C). This is also noticeable from the centred LRFs (Fig 5D). For example CR-spread-19m has significantly higher ML values than all other conditions and lower Θ values than most, and CR-night-2h-6m has significantly lower ML values and higher Θ values than all but CR-night-2h-19m (Fig 5). However, overall the ML values are relatively high and therefore confirm the observation that, although the timing can be displaced, the test data is close in data space to the training clock. This is compatible with the hypothesis that the different feeding condition induce a simple phase change in the clock.

Given these timing displacements, we carried out a comparison of the clocks under the different conditions by analysis using PDP plots where we regressed the phases of the genes against the timing displacements of the various conditions to try and quantify the extent to which the observed timing differences are the result of a coherent phase adjustment of each gene (Fig 5F). For the feeding×age conditions the situation is very clear for the core clock genes considered because the *r*^2^ values for them (Fig 5F) are typically close to 1 implying the linear model almost completely explains the data. From this analysis, we conclude that it is likely that the different feeding×age conditions cause a change in the core clock that is primarily a simple phase change and that for some of the conditions such as CR-day-2h this is substantial.

In summary, for this data, TimeTeller has enabled the discovery of substantial and coherent differences of the core clock systems state associated to the feeding conditions and provided quantified evidence that the core clocks corresponding to the different conditions differ by a simple phase change. This benefitted from a systems approach. Finally, note that although there is time series data in this instance, since our results on the test data samples are independent of each other having a time series is not necessary and also one could reduce the number of mice involved. This opens the possibility to use TimeTeller as a tool to determine a clock parameter in available QTL studies for longevity and other parameters [53].

#### Timing divergences and clock comparisons for the Mure *et al*. baboon data [2]

We found a different result when we compared the clocks in the different tissues studied in Mure *et al*. [2]. In this paper, the transcriptomes of 64 tissues of the diurnal primate *Papio anubis* (baboon) were analysed from one animal every 2 hours for 24 hours. The results of [2] demonstrate that many ubiquitously expressed genes that participate in essential cellular functions show a tissue-specific rhythmic pattern, and confirmed a shifted temporal organization of central and peripheral tissues between diurnal and nocturnal mammals. Since this RNA-seq dataset involves a genetically heterogeneous population and multiple transcriptionally heterogeneous tissues, we were keen to assess how well TimeTeller was able to analyse it.

We studied 33 of the tissues leaving out those from the brain and some others with missing data. An initial leave-one-tissue-out analysis gave reasonably accurate timing (MdAE around 1.23h, Fig 5G and Table 1) and indicated that many tissues had a substantial timing displacement (Fig 5H and 5J) ranging from approximately -3.5h to +2.5h compared to the time the samples were taken. The standard deviation of the individual sample apparent timing errors around the timing displacement from a given tissue was generally much smaller than the 6h range of the timing displacements (Fig 5H). Moreover, the null hypothesis that the *m*th most advanced tissue has the same timing displacement as the *m*th most retarded is rejected at the *p* = 0.01 level for all *m* < 7 (Wilcoxon-Mann-Whitney test).

Given many tissues had large absolute timing displacements, we then used only the 18 tissues with the smallest for the training data. This gives slightly better timing results than using all 33 tissues as can be seen in Table 1. Correcting the TimeTeller time predictions by adjusting them using the phase displacements of the tissues resulted in a substantial improvement of about half an hour in the timing accuracy (Fig 5G and Table 1). Given the heterogeneities in the data this results in a very reasonable performance with a mean absolute error of just over one hour.

The analysis of the variation of the core clock across the 64 tissues in Mure *et al*. [2] is mainly concerned with the overall transcript abundance and rhythmicity of expression of the individual core clock genes. The authors note that the heterogeneity of this implies different composition of core activators, repressors, and modulators in different tissues. They do not mention the timing divergences we find in the data using TimeTeller. Using these timing divergences, for the limited set of 33 tissues, we can study this in a different and more integrated way.

As above, we considered a comparison of the clocks in the different tissues by using a PDP plot (Fig 5K). For this dataset we see that the observed differences between them are not due to a simple coherent phase adjustment in the genes but involves a more complex interaction. This is because the *r*^2^ values, which measure of the proportion of total variation of outcomes explained by the linear model, are very low and much lower than those for the Bjarnason *et al*. [30] and Acosta-Rodríguez *et al*. data [37]. This suggests that the adjustment of the clock from tissue to tissue is more complex than a simple phase shift in the core clock genes. On the other hand, the relatively low *p*-values suggest that there is a definite correlation between gene phase and timing displacement suggesting that an appreciable component of the changes in the genes is a phase change.

Again this analysis benefitted from a systems approach which enables us to identify coherent differences between tissues and relate this to changes in the core clock.

### Probing the effect of changes in the core clock on downstream genes

Changes in the core clock will affect the regulation of rhythmic genes that are downstream of it. Current methods allow one to check whether these genes remain rhythmic when the clock is perturbed in some way but TimeTeller also allows examination of the extent to which they maintain their relationship with the clock in a coherent fashion. The way in which the different conditions of the Acosta-Rodríguez *et al*. mouse data [37] changed the phase of the core clock provides a very interesting example where we can demonstrate such an analysis.

Firstly, we noted that for the genes in the REP, all clock genes displayed approximately linear phase changes while for the other genes (*Hlf*, *Wee1* and *Cys1*) this was not the case for *Cys1*. We then used this analysis to look at the effect of the clock phase changes upon some other genes that are rhythmic in the liver of AL fed mice. In particular, we inspected the plots for some cell cycle genes and also a number of the genes identified in Acosta-Rodríguez *et al*. [37] as affected by the CR conditions or ageing. We find that inspection of the PDP plot for these genes gives clear and significant insight into the level of this coherence which we quantify by the *p*-value and *r*^2^ of the PDP plot.

Of the cell cycle genes *Wee1, p21, P53, Timeless, CyclinA, CHK2, CyclinB1, CyclinE2* and *ATM*, it appears that only *Wee1, p21* and *CyclinE2* are rhythmic in the liver in the AL conditions. These three genes maintain coherence with the clock under the other conditions with *Wee1* doing so strongly (*r*^2^ = 0.96) followed closely by *p21* (*r*^2^ = 0.92). The coherence of *CyclinE2* seemed somewhat weaker (*r*^2^ = 0.59). All of the other genes had *r*^2^ < 0.4 and appeared incoherent. There is a very strong correlation between the maintenance or absence of coherence and rhythmicity or non-rhythmicity.

In Acosta-Rodríguez *et al*. [37] a number of genes that were affected by ageing or the CR conditions were highlighted and sorted these into four categories: those susceptible to ageing-related changes under any condition tested, those related to fasting conditions, timing related genes and genes associated with effects on circadian cycling such as rhythmic damping. Our analysis using PDP plots for these genes clearly identifies which of them move coherently with the core clock under the different feeding conditions. None of the timing related genes stayed coherent and, amongst the fasting genes, only *Hal1* (*r*^2^ = 0.66) was. Several ageing genes show some level of coherence (Fig N in S1 Appendix) *Serpine1* (*r*^2^ = 0.66) *Adora1* (*r*^2^ = 0.68) *Got1* (*r*^2^ = 0.65) *Lepr* (*r*^2^ = 0.68) *Pfkfb5* (*r*^2^ = 0.88). For the genes affecting circadian cycling. while *Gys1* (*r*^2^ = 0.15) and *Per1* were incoherent, the rest were coherent: *Arntl* (*r*^2^ = 0.99), *Nr1d1* (*r*^2^ = 0.70), *Per1* (*r*^2^ = 0.69), *Per2* (*r*^2^ = 0.97) and *Pck1* (*r*^2^ = 0.60). For a significant number of the genes affected by ageing or the CR conditions, while the gene is not coherent under all conditions it is coherent under a significant number of the conditions with the less extreme timing deviations. This can be seen from the PCPs and was the case for *Per1* which seems coherent under all conditions except the four CR-day conditions.

These results demonstrate that such an analysis can give a novel overview of gene response and whether a given gene maintains coherence with the clock when the clock timing changes. Such coherence is associated with genes that show good linearity with a significant slope in the PDP plots. Consequently, TimeTeller can be used to investigate function and dysfunction in genes controlled by the circadian clock when the clock is perturbed.

## Methods

### Ethics statement

The study associated with the Bjarnason *et al*. human data [30] was approved by the Sunnybrook Health Sciences Centre Research Ethics Board. Project identification number 396–2004. Written informed consent was obtained from each subject as requested by the Research Ethics Board.

### Probability model constructed from training data

The training data will have been collected at sample times *t*_*i*_, *i* = 1, …, *N*_*t*_. In the training data used here the number *N*_*s*_ of samples at each time point is the same. Therefore, if the samples are indexed by *j*, the *G*-dimensional REVs with sample time *t*_*i*_ can be labelled by *i* and *j* and denoted ${\bar{g}}_{ij}$.

In three of the training datasets the *instances* *j* correspond to different tissues (with replicates in one case) and in the other (Bjarnason *et al*. [30]) to different individuals. Each *g*_*ij*_ is then normalised using timecourse and/or intergene normalisation as described above resulting in vectors $g_{ij}^{\text{norm}}$ that will be used to train TimeTeller. The issue of batch effects is considered in Note A in S1 Appendix.

To construct the probability model we firstly construct one for each timepoint *t*_*i*_ in the training data by using the local statistical structure of the data at that timepoint and then we combine these. Associated with this time *t*_*i*_ is the set $D_{i}$ of *N*_*s*_*G*-dimensional vectors $g_{ij}^{\text{norm}}$, *j* = 1, …, *N*_*s*_. We calculate the principal components *U*_*i*,*k*_ of this dataset and then use the first *d* of these to define a projection *P*_*i*_ of the normalised training data into $R^{d}$ (Note F in S1 Appendix) i.e. $P_{i}\left(g\right)=U_{d}^{T}\cdot g$ where *U*_*d*_ is the matrix made up from the column vectors *U*_*i*,*k*_ for *k* = 1, …, *d*. We then fit a multivariate normal distribution (MVN) $P_{i,j}$ to the points $P_{i}\left(D_{j}\right)$. The dimensionality *d* is chosen so that there are enough vectors in $P_{i}\left(D_{j}\right)$ to fit a *d*-dimensional multivariate Gaussian (using the MATLAB function fitgmdist) while ensuring that most of the variance in the data is captured by the *d*-dimensional projection (e.g. see Fig F in S1 Appendix). In our case we take *d* = 3.

Now we fix a time *t*_*i*_ and consider the means *μ*_*j*_ and covariance matrices Σ_*j*_ of the distributions $P_{i,j}$. We fit a periodic piecewise cubic hermite interpolating polynomial spline through the *μ*_*j*_ and each of the *d*(*d* + 1)/2 entries that determine Σ_*j*_ so as to extend *μ*_*j*_ and Σ_*j*_ to all times *t* between the time points checking that the Σ_*j*_ are positive definite and moving them to the nearest positive definite matrix if this is not the case. We thus obtain *μ*_*i*_(*t*) and Σ_*i*_(*t*) and thus the associated family of *d*-dimensional MVN distributions $P_{i,t}$ for all times *t* between the first and last data times. For these splines we use the MATLAB function *perpchip* as this respects the periodicity in *t*. Our implementation offers some alternatives to *perpchip* but these are not used here. This family of MVN distributions indexed by time is what we refer to as the *probability model*.

### The likelihood curve *L*_*g*_(*t*) and the log threshold *l*_thresh_

Now we define the likelihood curve *L*_*g*_(*t*) where *g* is a REV from either training or test data. Having calculated the probability model, for a given REV *g*, for each of the time indices *i* we define the likelihood curve associated with the *i*th timepoint using the probability given by the MVNs $P_{i,t}$ i.e. $L_{g,i}\left(t\right)=P_{i,t}\left(g^{\text{norm}}\right)$ where *g*^norm^ is the vector obtained after normalising *g* with the relevant normalisation.

The idea is to obtain log *L*_*g*_(*t*) by averaging these individual log likelihoods log *L*_*g*,*i*_, *i* = 1, …, *N*_*t*_ but some modification is needed. We will need to fix a lower threshold *l*_thresh_ < 0 and replace each log *L*_*g*,*i*_ by max{log *L*_*g*,*i*_, *l*_thresh_} in the sum so that *L*_*g*_(*t*) is defined by $\text{log}\;L_{g}\left(t\right)=N_{t}^{-1}\sum_{i=1}^{N_{t}}\text{max}\left\{\text{log}\;L_{g,i},l_{\text{thresh}}\right\}$.

This truncation is necessary to ensure that this sum is not wrecked by inaccurate exceptionally low values of one *L*_*g*,*i*_ affecting robust high values of another at the same *t*. A curve *L*_*g*,*i*_ may take on very low values away from its maximum and the exact values of these very low probabilities may well be unreliable and inaccurate. If this happens at a *t* value for which another such curve *L*_*g*,*j*_ has a high accurate value then this may badly affect the estimate of *L*_*g*_(*t*). The way to choose the value of *l*_thresh_ is discussed in Note C in S1 Appendix.

### Definition of Θ

The *clock dysfunction metric* Θ is defined to be the proportion of time *t* where the LRF is greater than *C*(*t*|*T*) = *η*(1 + *ϵ* + cos 2*π*(*t* − *T*)/24) which is a scaled cosine function phase shifted so that the maximum is at *T* (Fig 2D). The parameters must satisfy 0 < *ηϵ* < *η*(2 + *ϵ*) < 1. Although we have experimented with changes, the effect on Θ of changing *η* and *ε* is clear (see Fig 2D) and we have seen no reason for changing them from the values we have used here.

### Choice of parameter *l*_thresh_

The key considerations underlying the choice of *l*_thresh_ are that it should be as large as possible subject to the conditions that (i) very few training and control samples have flat regions that significantly intersect *C*(*t*|*T*) so that they contribute significantly to Θ, and (ii) as many as possible of the test data samples should have MLs above *exp*(*l*_thresh_).

There are two reasons we do not want *l*_thresh_ to be decreased further than necessary. Firstly, the considerations above about the need to protect against inaccurate exceptionally low values of some *L*_*g*,*i*_ and, secondly, because if *l*_thresh_ is reduced too far structure in the LRF at times that are away from the time *T* is likely to be removed. This happens because, if the *L*_*g*,*i*_ have their maxima not too far from *T* then decreasing *l*_thresh_ causes a much bigger decrease in the likelihood *L*_*g*_(*t*) for *t* away from *T* than near to *T* and therefore decreases the LRF away from *T* while maintaining the peak structure near *T*. If the dysfunction is mainly manifested by low ML then decreasing *l*_thresh_ by too much moves all the flat regions in the LRFs down below *C*(*t*|*T*) while if it manifested by structures such as second peaks then these are also decreased below *C*(*t*|*T*). In both cases this results in a decrease in Θ. These phenomena are illustrated in Note C and Fig H in S1 Appendix.

If the maximum value log ML of log *L*_*g*_(*t*) is only just above *l*_thresh_, then it and the corresponding likelihood ratio curve will have intervals on which they are flat. If this is the case then the length of these flat intervals above the minimum of the curve *C*(*t*|*T*) can contribute to Θ. This contribution has interesting information in it because it is related to how low the maximum value ML of *L*_*g*_(*t*) is.

If the criterion (ii) results in too small a value so that too much structure has been removed, it is then generally acceptable to set *l*_thresh_ at a higher value provided that the number of training samples violating (i) does not get too large. It so also desirable that the the number of test samples violating (ii) is not too large as otherwise many samples have Θ = 1 meaning that these samples do not have a non-trivial stratification even though they will be distinguished as having higher dysfunction than other samples.

## Discussion

What we hope stands out is the way TimeTeller can be used to study single samples of external test data in ways that reach beyond the information provided by current algorithms. The main aim of this study was to indicate the different ways that TimeTeller can be used to visualise and probe the circadian clock as a system.

Understanding internal timing *T* is important because, for example, a patient’s phase shift is critical for guiding personalised timing of chronotherapy but our fundamental assertion is that the TimeTeller likelihood curve contains more information about clock dysfunction than just timing. We believe that the examples we discuss bring this out. The algorithm’s output is not just limited to the timing estimate alone but also comes with an estimate of Θ, ML and the likelihood curve. Thus, one has much more information with which to assess both dysfunction and the assessment’s quality.

We give many examples where the dysfunction metrics Θ and ML that we introduce take statistically significant different values in perturbed conditions compared to WT/control. An important aspect of this analytical approach is that Θ can provide a stratification of individual transcriptomes by measured dysfunction. This is important because it enables the possibility of associating clock dysfunction with other aspects of disease on the level of the individual. This is illustrated most clearly by our analysis of the Boyle *et al*. data [43] on the effects of smoking on the transcriptome of the human oral mucosa and that of Feng *et al*. data [50] on oral squamous cell carcinoma. This analysis showed significant differences between the smokers and non-smokers in the Boyle *et al*. data [43] and between normal and cancer for the Feng *et al*. data [50] and in both cases enabled the identification of a “bad clock” group with a significant number of differentially expressed genes compared to other individuals of the same cohort (smoker or cancerous tissue).

When analysing the cancer data samples from Feng *et al*. [50] and Cadenas *et al*. [51] we were able to validate the quality of timing estimates without using any time stamps. This means that we were able to identify a large number of patients with significant dysfunction in the clock but still identify the sample time which for the Feng *et al*. data [50] often involved the second peak in the LRF. Moreover, this method of analysis gives a new way to estimate the precision of timing/phase algorithms on large data sets even if they are not time-stamped and even if they contain significant dysfunction as is the case with the Feng *et al*. data [50]. In a future paper we expect to apply TimeTeller to study other cancer datasets.

TimeTeller offers other new possibilities for the analysis of timeseries data as shown by the analysis of the Bjarnason *et al*. [30], Acosta-Rodríguez *et al*. [37] and Mure *et al*. data [2]. Firstly, TimeTeller allowed us to identify significant timing displacements for the individuals, conditions or tissues that had not been observed and it was not necessary for these data to be in time-series. Secondly, when these are in time series, by identifying the timing displacements and then regressing the gene phases against them, we were able to compare the clock in different individuals, conditions or tissues and attempt to assess whether the difference is largely a phase shift or a more complex adjustment. Moreover, we show how to analyse genes downstream of the clock in a similar way. For example, using the Acosta-Rodríguez *et al*. mouse data [37] we were able to see which genes maintained their rhythmicity and coherence with the clock in all the temporally restricted feeding conditions and which did not.

Because TimeTeller’s results on test data samples are independent of each other having a test time series is not necessary and this suggests that use of TimeTeller might facilitate a reduction in the number of animals involved.

An important insight of the study of Wittenbrink *et al*. [23] is the need to develop optimised high-quality data that is cheap to collect. This will also be important for the use of TimeTeller. While it is clear that the sort of data we discuss in this paper will become increasingly abundant and much cheaper to generate, other data types such as Nanostring’s nCounter platform [54] might be more suitable to clinical workflows and may be used to provide cheaper purpose-designed datasets that can be used with TimeTeller. This will also bring the opportunity to improve TimeTeller because timecourse normalisation will be less necessary and the training will be improved by having more training data at more time points around the day.

The algorithm is very customisable and flexible and relatively fast. For example, on a Macbook Pro (2021) with an 8-core M1 chip and 16Gb of memory, calculation of the probability models takes between 3 and 5 seconds and the leave-one-out analysis of the training datasets take an average of between 0.71 and 0.76 seconds for test data analysis of a sample. The user is free to choose the genes employed by TimeTeller and experiment with the parameters *l*_thresh_, *η* and *ε*. Although we have experimented with changes, the effect of changing *η* and *ε* is clear from Fig 2A and 2D and we have seen no reason for changing them from the values we have used here. Keeping them constant means that Θ values can be compared across datasets.

On the other hand, *l*_thresh_ needs to be chosen using the data for the reasons explained in Methods and Note C in S1 Appendix. While the value of *l*_thresh_ will vary with different transcriptomic platforms and experimental protocols in a situation where new test data is arriving in multiple batches it should not be the case that *l*_thresh_ is constantly being reassessed. A consensus value should quickly be arrived at. we believe that in its use, for example, with individuals in a clinical context it will be possible to settle on a platform and protocol and value of *l*_thresh_ that can be used across all test data.

## Supporting information

S1 Appendix Notes, Figures, Tables. (PDF)
