# Supplementary material for: TimeTeller: A tool to probe the circadian clock as a multigene dynamical system
Source: PLoS Comput Biol. 2024 Feb 29;20(2):e1011779. doi: 10.1371/journal.pcbi.1011779 (PMC10931517; doi:10.1371/journal.pcbi.1011779)
Supplement: S1 Appendix — (PDF) [file pcbi.1011779.s001.pdf]

Supplementary Material for the paper "TimeTeller: a tool to probe the circadian clock as a multigene dynamical system"

Denise Vlachou[1a], Maria Veretennikova[1b], Laura Usselmann[5c], Vadim Vasilyev[5]
Sascha Ott[5], Georg A. Bjarnason[2], Robert Dallmann[5] Francis Levi[3,5] & David A. Rand[1,3*]

[1]Mathematics Institute & Zeeman Institute for Systems Biology.
and Infectious Epidemiology Research, University of Warwick, Coventry CV4 7AL, UK.
[2]Odette Cancer Centre, Sunnybrook Health Sciences Centre, 2075 Bayview Ave.,
Toronto, ON, M4N 3M5, Canada.
[3]European Associated Laboratory "Personalizing Cancer Chronotherapy through Systems Medicine",
Institut National de la Santé et de la Recherche Médicale, UMRS 935,
Campus CNRS, 7 rue Guy Moquet, 9480- Villejuif, France.
[4]Assistance Publique-Hopitaux de Paris, Saint-Louis Hospital, Breast Disease Unit,
University Paris Diderot, 75475 Paris, France.
[5] Division of Biomedical Sciences, Warwick Medical School, University of Warwick, Coventry CV4 7AL, UK.

*To whom correspondence should be addressed;
E-mail: D.A.Rand@warwick.ac.uk.

[a]Current address: GSK Research, Gunnels Wood Road, Stevenage, Herts, SG1 2NY,UK
[b]Current address: CAMS Oxford Institute, Nuffield Department of Medicine, University of Oxford, Old Road Campus, Oxford, OX3 7BN
[c]Current address: Hit Discovery, Discovery Sciences, R&D, AstraZeneca, Cambridge, CB2 0AA, UK.

Part I

Supplementary Notes

Throughout these notes the main paper is referred to as **I**.

Note A. Data sets

Mouse training datasets

Mouse training datasets	
**Zhang et al. 2014 Microarray (*1*)**	
Technology	Microarray (Affymetrix MoGene 1.0 ST arrays)
GEO	GSE54652
Tissue(s)	Adrenal, aorta, brown fat, heart, kidney, liver, lung, skeletal muscle, white fat
Entrainment conditions	1 week 12 hr light/ 12 hr dark. Sample collection commenced after 18 hrs constant darkness.
Experimental conditions	Wild type.
Timepoints	CT18 - CT64, every 2 hrs.
Number of replicates	1 sample per timepoint consisting of 3 pooled mice
Male or female?	Male
Age	6 weeks
Strain	C57BL/6
**Zhang et al. 2014 RNA-seq (*1*)**	
Technology	RNA-seq
GEO	GSE54652
Tissue(s)	Adrenal, aorta, brown fat, heart, kidney, liver, lung, skeletal muscle, white fat
Entrainment conditions	1 week 12 hr light/ 12 hr dark.
Experimental conditions	Sample collection commenced after 18 hrs constant darkness. Wild type
Timepoints	CT22 - CT64, every 6 hrs.
Number of replicates	1 sample per timepoint consisting of 3 pooled mice
Male or female?	Male
Age	6 weeks
Strain	C57BL/6

Mouse test datasets

Mouse test datasets	
**Kinouchi et al., 2018 RNA-seq** (*2*)	
Technology	RNA-seq
GEO	GSE107787
Tissue(s)	Liver, skeletal muscle
Entrainment conditions	12hrlight/12hrdark
Experimental conditions	Ad libitum fed vs 24 hr starved
Timepoints	ZT0 -ZT20, every 4 hrs.
Number of replicates	3 samples per timepoint
Male or female?	Male
Age	8 weeks
Strain	C57BL/6
**Weger et al., 2021** (*3*)	
Technology	RNA-seq
GEO	GSE135898
Tissue(s)	Liver
Entrainment conditions	12hrlight/12hrdark
Experimental conditions	*Bmal* KO with WT controls and Cry1/2 double KO with WT
Timepoints	controls ZT0 - ZT20, every 4 hrs.
Number of replicates	2 samples per timepoint
Male or female?	Male
Age	12-16 weeks
Strain	C57BL/6
**Yeung et al., 2018 (*1*)**	
Technology	RNA-seq
GEO	GSE100457

Mouse test datasets (cont'd)	
Tissue(s)	Kidney
Entrainment conditions	12 hr light / 12 hr dark
Experimental conditions	*Bmal* KO with WT controls. Night restricted feeding protocol for all mice.
Timepoints	ZT0 - ZT20, every 4 hrs.
Number of replicates	2 samples per timepoint
Male or female?	Male
Age	8-12 weeks
Strain	C57BL/6
**Fang *et al.*** (*4*)	
Technology	Microarray (Affymetrix MoGene 1.0 ST arrays)
GEO	GSE59460
Tissue(s)	Liver
Entrainment conditions	12 hr light / 12 hr dark
Experimental conditions	5 Wild Type and 5 *NR1D1* KO mice
Timepoints	ZT10
Number of replicates	1
Male or female?	Male
Age	8-12 weeks
Strain	C57BL/6
**Barclay *et al.*** (***5***)	
Technology	Microarray (Affymetrix MoGene 1.0 ST arrays)
GEO	GSE33381
Tissue(s)	Liver and adipose samples
Entrainment conditions	12 hr light / 12 hr dark
Experimental conditions	Half of mice were were kept awake during ZT 0-6 on days 1 - 5 and days 8 - 12. Fed *ad libitum*
Timepoints	ZT1, ZT7, ZT13 and ZT19
Number of replicates	3
Male or female?	Male
Age	8-12 weeks
Strain	C57BL/6
**LeMartelot *et al.*** (***6***)	
Technology	Microarray (Affymetrix MoGene 1.0 ST arrays)
GEO	GSE35789
Tissue(s)	Liver, pooled from 5 mice
Entrainment conditions	12 hr light / 12 hr dark
Experimental conditions	WT
Timepoints	ZT2, ZT6, ZT10, ZT14, ZT18, ZT22, ZT2(+24)
Number of replicates	1 pooled from 5 mice
Male or female?	Male
Age	8-12 weeks
**Acosta-Rodríguez *et al.*** (*7*)	
Technology	RNA-seq
GEO	GSE190939
Tissue(s)	Liver
Entrainment conditions	Constant darkness
Experimental conditions	WT mice in 6 feeding conditions
Timepoints	12 time points
Number of replicates	2 (these are the observations at t and t+24)
Male or female?	Male
Age	6 months & 19 months
Strain	C57BL/6J
**Koronowski *et al.*** (*8*)	
Technology	RNA-seq
GEO	GSE117134
Tissue(s)	Liver
Entrainment conditions	12hr light/ 12hr dark schedule
Experimental conditions	wild type (WT), *Arntl* knockout (KO), Liver-RE-Arntl-stop-FL (reconstructed)
Timepoints	ZT00, ZT04, ZT08, ZT12, ZT16, ZT20
Number of replicates	3
Male or female?	Female
Age	8-12 weeks
Strain	C57BL/6J

Baboon training dataset from Mure *et al.* (*9*)

Baboon training dataset	
**Mure et al. 2014 RNA-seq (*9*)**	
Technology	RNA-seq
GEO	GSE98965
Tissue(s)	Adrenal cortex, Adrenal medulla, Antrum, aorta, axillary, bladder, bone marrow, cecum, cornea, heart, ileum, kidney cortex, kidney medulla, liver, lungs, mesentric lymphonodes muscle abdominal, muscle gastrocnemius, oesophagus, omental fat, optic nerve head, pancreas, prostate, retina, retinal pigment epithelium, skin, smooth muscle, spleen, white adipose mesenteric, white adipose pericardial, white adipose perirenal, white adipose subcutaneous, and white adipose tissue.
Entrainment conditions	12 hr light/ 12 hr dark.
Experimental conditions	Sample collection commenced after 18 hrs constant darkness. Wild type
Timepoints	ZT00 - ZT22, every 2 hrs. where ZT00 is time light is switched ON, ZT12 is time light is switched OFF (and no food intake)
Number of replicates	1, all tissues from a baboon sampled per timepoint
Male or female?	Male
Age	young
Strain	C57BL/6

Human training dataset

Human: Oral Mucosa Timecourse microarray data (Bjarnason *et al.*)

For this study Bjarnason *et al.* (*10*) recruited ten healthy human volunteers, five female and five male. Mucosa tissue was collected at six time points: 8 am, noon, 4 pm, 8 pm, 12 midnight, and 4 am. Subjects were selected after screening by clinical history, physical examination, routine blood work (complete blood count, electrolytes, creatinine) and actigraphy to confirm regular sleep-wake patterns. Mucosa samples were collected by a dental surgeon, using a tissue punch biopsy. Subjects went to sleep in a darkroom at their usual bedtime and were awoken for the midnight and the 4 am samples[a].

After collection, tissue mucosa samples were immediately frozen in liquid nitrogen and stored at $-80°C$ until use. Total RNA was prepared by Trizol Reagent (Invitrogen) in accordance with the manufacturer's specifications. RNA samples were quantified by optical density measurements at A260nm and A280nm. All samples were determined to be of high quality with A260:A280 ratios $>$ 1.9. Total RNA (5μg) of each sample was used for microarray analysis on Affymetrix HG_U133_Plus2 chips. Cumulatively this chip represents 54,679 gene transcripts for analysis. Biotinylated cRNA was prepared according to the standard Affymetrix protocol (Expression Analysis Technical Manual, 2004, Affymetrix). Following fragmentation, 15 μg of cRNA were hybridized for 16 hrs at $45°C$ on GeneChip Human Genome U133 Plus 2.0 arrays. GeneChips were washed and stained in the Affymetrix Fluidics Station 450 and were scanned using the Affymetrix GeneChip Scanner 3000.

Although there were 16 probes identified in Note Fig B. as rhythmic and synchronised, we only use 15 probes going forward. The reason is that the *Per1* probe 244677_at was found to have significant signal issues in many of the independent human datasets, i.e. the signals values were very low. As there is another *Per1* probe in this dataset that does not have this problem, we can conclude that this is a probe issue, and not an issue with the *Per1* gene expression.

The study was approved by the Sunnybrook Health Sciences Centre Research Ethics Board. Project identification number 396-2004. Written informed consent was obtained from each subject as requested by the researchers ethics board.

[a]The Research Ethics Board at Sunnybrook Health Science Centre approved the clinical protocol for this study.

Human test datasets

Human test datasets	
**Boyle *et al.* (*11*)**	
Technology	GeneChip Human Genome U133 Plus 2.0 arrays
GEO	GSE59460
Tissue(s)	Oral mucosa
Samples	40 current smokers and 40 age and gender matched never-smokers underwent buccal biopsies. One smoker sample was excluded from the study based on a quality measure.
Study	Effects of smoking on the oral mucosal transcriptome
Timepoints	Unknown
Number of replicates	1
Gender	Mixed, gender matched
**Feng *et al.* (*12*)**	
Technology	GeneChip Human Genome U133 Plus 2.0 arrays
GEO	GSE59460
Tissue(s)	Oral mucosa
Samples	229 samples in total, 167 of OSCCs, 45 of normal oral mucosa, and 17 samples are of dysplasic oral mucosa tissue
Sudy	a comparative analysis of healthy oral mucosa transcriptome and oral squamous cell carcinoma (OSCC) transcriptome
Timepoints	Unknown
Number of replicates	1
Gender	Mixed
Note	Dysplasic tissue is abnormal tissue that could signify early signs of cancer.
**Cadenas *et al.* (*13*)**	
Technology	Affymetrix HG-U133A arrays
GEO	GSE11121, GSE2034, GSE6532 and GSE7390
Tissue(s)	Breast cancer tumour
Samples	Mainz n=200, Rotterdam n=286, Transbig n=280. Total 766.
Study	A study of the association of clock gene expression with tumor progression in breast cancer
Timepoints	Unknown
Number of replicates	1
Note	Metadata, details and analysis is in Schmidt *et al.* . Cancer Res 2008; 68:5405-13; PMID:18593943.

Preparatory RNAseq methods.

All RNA-seq datasets were downloaded as .sra files from NCBIU's SRA database (accessed via the GEO database). SRA files were converted to fastq files using "fasterq-dump" from NCBIU's SRA Toolkit. FASTQ files were aligned to the mouse genome (GRC release m38.84) and converted to SAM files using HISAT2 v2.2.0 (Kim et al., 2019). SAM files were compressed to BAM using Samtools v1.10 (Heng Li et al., 2009). Transcript read counts were determined from the BAM files and the mouse transcriptome (GRCm38.84 .gtf file) using LiBiNorm v2.4, an in-house software package, in HTSeq-count mode (Anders et al., 2015; Dyer et al., 2019). Raw read counts were concatenated for all samples and exported as one text file for all subsequent analysis on a Mac OS. For dataset specific arguments see Table 4.2. Raw count normalisation was carried out in R Studio. The edgeR package was used to normalise the raw counts to log2 counts per million (logCPM) or log2 trimmed mean of M-values (logTMM). Data was also inspected for quality by checking library sizes, replicate plots and PCA plots.

Preparatory microarray methods.

The raw data was downloaded from NCBI GEO in the form of 288 .CEL files. The bioconductor package in R was used to perform fRMA normalisation of protein coding genes, and annotate them

with gene names. After fRMA processing, the gene expression values are expressed in log2, and values are in the range 2-14. The distribution of gene expression values for all 288 sets, summarising 35,556 probes was analysed to verify similar distribution amongst samples and to check that the fRMA normalisation was successful.

Batch Effects

In high-throughput studies batch effects occur because measurements are affected by variations in experimental conditions such as laboratory conditions, reagent lots, and personnel differences. We used the R packages BatchQC, ComBat and sva to analyse batch effects.

For the training data we are only concerned with the $G = 9 - 16$ selected clock-associated genes. We inspected the embedding of the data into G-dimensional space and the subsequent projections into 3-dimensional space using SVD for any sign of batch effects especially with respect to timing of the samples (see e.g. SI Fig S4). Following analysis, the timing, Θ and ML values, and the likelihood ratio curves are inspected for signs of batch effects where this is relevant. A similar analysis is carried out after TimeTeller normalisation, a process that also reduces batch effects. We want to avoid batch correction as this would arbitrarily destroy the statistical distribution of the data (e.g., the covariance information) which is a crucial ingredient of our algorithm.

For the test data there is no question of a batch effect affecting the determination of Θ in a test data set as each sample is treated individually i.e. it is treated as an independent single sample in a way that does not depend upon any other test samples under both TimeTeller normalisation and the initial fRMA normalisation and then the probability model (which only depends upon the training data) is applied to the data from this sample independently. Our analysis also found no evidence of batch effects in this data when projected into $d = 3$-dimensional space using PCA and when analysed as above.

Using algorithms such as ComBat to remove batch effects between different tissue types has several disadvantages. Firstly, batches will typically not be evenly balanced as the training data will only contain WT/healthy data, whilst test data batches will contain both WT/healthy and perturbed data (e.g., genetically manipulated knock-out (KO) samples). The inappropriate application of batch correction methods to datasets with unbalanced batches is reviewed elsewhere (*14*). A second disadvantage to the use of batch correction is that it necessitates that the model be re-trained for every new training and test dataset combination post-batch correction, therefore it is difficult to directly compare findings from different test datasets. Ultimately, one of the primary aims is to apply TimeTeller to human biopsies - both healthy and unhealthy. Batch correction of human data would require *a priori* knowledge of covariates, which may not even be known. In other words, 'real world' data contains many variables which would confound batch correction such as patient age, sex, time of sampling, health status etc. Therefore, a method for time prediction should ideally be applicable without the need for batch correction.

Data availability

All data is currently publically available except for the Bjarnason *et al.* data used in the paper. This will be made available on publication via the University of Warwick servers and as part of the R package that will be available via GitHub.

Note B. Does the rhythmic expression profile (REP) provide a faithful representation of clock dynamics?

It is necessary to try and ensure that the choice of the the REP and rhythmic properties of the genes included provide a faithful representation of the clock state i.e. so that the mapping from clock state to NREV $(g_1, \ldots, g_n)$ is an embedding. This means that the NREV is a smooth function of the clock state and that each state of the NREV arises from at most one clock state. Conditions for this to be

the case are discussed in the area of applied dynamical systems known as *embedology* (*15–17*). This addresses the situation where one has a dynamical system whose state depends upon many variables $y_1, \ldots, y_N$ and one seeks a faithful representation of the dynamics in terms of a much reduced number of variables $x_1, \ldots, x_n$ and shows that for oscillating non-chaotic systems like ours it is reasonable to find such a representation by the sort of projections we use. In our case the y_i represent the expression levels of all the molecular components of the GRN underlying the clock and the x_i are the expression levels of the genes in the REP. There is no way to prove that such a REP does fully represent the dynamics but for a tightly coupled dynamical system like the circadian clock, one can validate this to a great extent by checking that increasing the size of the REP does not alter results and, more specifically, checking the singular values of the local projections introduced in Methods as in SI Fig S3 to ensure that the dynamics of the n-dimensional REP can be reduced to an even smaller dimension d as explained in Methods. We deal with it by the use of a REP with significantly more genes than are likely to be needed. Note that we are not suggesting that the genes in the REP are the only genes regulating the clock but are hypothesising that they are enough to provide a full representation of the clock dynamics for the data sets considered.

Note C. Choice of l_{thresh}.

We discuss how the cut-off threshold l_{thresh} is chosen using the example of the two Kinouchi *et al.* datasets, one for skeletal muscle and the other for and liver. It is also useful to consider SI Fig S6I which looks at how Θ changes as l_{thresh} is reduced from -5 to -12 for the Bjarnason *et al.* human data.

SI Fig S15 concerns the Kinouchi *et al.* skeletal muscle data. The first step is to get an estimate of the the values of ML. From Fig S4 Note we see the median of these for FAST samples from this data is a bit below 10^{-5} which is approximately $\exp(-11.5)$ which suggests a value of -10 or -12 for l_{thresh}. The three columns in SI Fig S15 Fig O. correspond to respectively $l_{\text{thresh}} = -10$, -12 and -14. The setting $l_{\text{thresh}} = -10$ is seemingly an acceptable value because then only one of the FED control data samples has a flat region that intersects $C(t|T)$. However, rather a lot of the test samples have their ML below e^{-10} so a smaller l_{thresh} of -12 or -14 is preferable. With $l_{\text{thresh}} = -12$ none of the FED samples intersects the curve $C(t|T)$ and this setting is what we have used in the main paper. D, E and F in SI Fig S15 show the corresponding maximum likelihood values. With this choice of l_{thresh} there are still some test data samples with their ML below $e^{l_{\text{thresh}}}$ so that they will have $\Theta = 1$. They are just identified as all having extreme $lowML$ dysfunction.

The MLs hardly change with changing l_{thresh} the key difference just concerns the change in the number of samples with their ML value at the value of the threshold $\exp(l_{\text{thresh}})$. This change occurs because there are FAST samples where ML is below the cut-off threshold and the number of these below-threshold samples decreases as we decrease l_{thresh}.

The Θ values that are obtained with these thresholds are shown in SI Fig S15 The way that the values change is shown in SI Fig S15 Fig. Fig O.J,K. We see that when we change from -12 to -14 there is hardly any change in the ordering of the Θ values and hence the stratification, and this further justifies a choice of $l_{\text{thresh}} = -12$.

This choice is for the skeletal data. However, when we analyse the Kinouchi *et al.* liver data using $l_{\text{thresh}} = -12$ we obtain the results shown in SI Fig S8. These suggest that the substantially worse timing for the FAST data is due to type $lowML$ dysfunction. However, all the structure in the FAST LRFs except for the central peak has been removed as discussed above and consequently the observed Θ values do not pick this $lowML$ dysfunction up. A quick inspection of the MLs suggests that $l_{\text{thresh}} = -12$ gives too low a threshold because only two sample have a ML below $\exp(-5.5)$ and these are much lower at approximately $\exp(-10)$. This suggests using a threshold with $l_{\text{thresh}} = -6$ or -7. In SI Fig S15 we used $l_{\text{thresh}} = -6$ and we see that then the Θ spectrum nicely picks up the $lowML$ dysfunction. One might ask why we did not just use the ML to stratify things in this case and, indeed, this would give essentially the same answer for this data. However, this is not true when our samples have significant structures like second peaks as, for example seen in the Fang *et al.* , Boyle *et*

al. and Feng *et al.* datasets and in other cancer datasets that we have studied. for these Θ integrates the different types of dysfunction.

Note D. Role of gene-to-gene correlations in the likelihood function

Θ contains an estimate of a direct measure of clock precision

In what follows $g = (g_1, \ldots, g_G)$ denotes a vector consisting of the possibly normalised levels of G clock-associated genes. We call these *normalised rhythmic expression vectors* (NREVs).

We consider the probability $P(t, g)$ that g is observed at time t in WT conditions and the associated conditional probability distributions $P(g|t)$ and $P(t|g)$. Then if we want to assess how well the clock is working in an independent sample with expression vector $g^* \in \mathbb{R}^G$ we would want to estimate the conditional distribution $P(t|g^*)$. The time T would be estimated to be the value of t maximising $P(t|g^*)$ i.e. the maximum likelihood estimate (MLE).

The corresponding likelihood ratio is given by

$$\Lambda(t) = P(t|g^*)/P(T|g^*).$$

As is well-known (e.g., Chaps. 8 & 9 of (*18*)) the likelihood ratio confidence interval gives a good estimate of the confidence intervals for the MLE when the log likelihood function is approximately quadratic on some scale as is the case with our likelihood functions. If α is the required sensitivity (i.e. 1 minus the confidence level) then the confidence interval is given by the set of times t that satisfy

$$\Lambda(t) \geq \exp -\chi^2_{\alpha,1}/2.$$

We can relate this to Θ (see Note S2) when the likelihood curve only has a single peak (or, more generally, only twice meets the curve C in Note S2). Since the likelihood ratio curve is quadratic near its maximum we deduce that the contribution to Θ from the region around the maximum is

$$\Theta = \sqrt{2\eta/\chi^2_{\alpha,1}}C$$

where C is the length of the confidence interval and η is the parameter in Methods. Since the confidence intervals define the precision of the clock we see that, in this case, Θ is a direct measure of clock precision.

$P(t|g)$ is estimated using $P(g|t)$

To measure $P(t|g)$ we will use the fact that, by Bayes theorem, so far as dependence upon t is concerned,

$$P(t|g) = \frac{P(g|T)P(t)}{P(g)} \propto P(g|t)$$

provided we assume that $P(t)$ is reasonably independent of t. Therefore, we can use $P(g|t)/P(g|T)$ to estimate $\Lambda(t) = P(t|g)/P(T|g)$. Consequently, TimeTeller tries to estimate $P(g|t)$ directly from WT data. For the estimation it is assumed that $P(g|t)$ is multivariate normal.

Θ depends crucially on the covariance structure of $P(g|t)$

To see what Θ depends upon consider the case where $P(t|g^*)$ is relatively sharply peaked around T. Expanding around this estimate, the distribution is approximately Gaussian with

$$P(t|g^*) \approx \frac{1}{\sqrt{2\pi\sigma_t}} \exp\left[\frac{(t-T)^2}{2\sigma_t^2}\right]$$

and the variance σ_t is given by

$$\frac{1}{\sigma_t^2} = \delta g^T \cdot \Sigma^{-1} \cdot \delta g$$

where Σ is the covariance matrix of $P(g|t)$ and δg is the derivative with respect to t of the mean of $P(g|t)$ at $g = g^*, t = T$. Indeed, if we drop the tightness assumption we can use the Cramer-Rao theorem to deduce that the term on the righthand side is a lower bound for the variance because this term is the dominant part of the Fisher Information matrix of $P(g|t)$ with respect to t.

Therefore, we see that it is crucial in estimating our clock dysfunction metric that we take account of the covariance structure of $P(g|t)$.

Note E. Singular Value Decomposition and projections

Suppose $\mathbf{v} = v_1, v_2, \ldots, v_N$ is a set of N n-dimensional data vectors and M is the $N \times n$ matrix whose columns are these vectors v_i. Let $M = UDV^*$ be the SVD of M ($*$ denoting transpose). Singular Value Decomposition (SVD) gives a decomposition of any $N \times n$ matrix such as M into a product of the form $M = UDV^*$ where U is a $N \times n$ column-orthonormal matrix ($UU^* = I_N$ and $U^*U = I_n$), V is a $n \times n$ orthonormal matrix and $D = \text{diag}(\sigma_1, \ldots, \sigma_n)$ is a diagonal matrix. The columns U_i of U are the principal components (PCs) and the ordered elements $\sigma_1 \geq \cdots \geq \sigma_n$ are the *singular values*. The projection of a vector v_i onto the first d PCs is given by

$$v_{i,d} = U^{[d]} v_i = \sum_{j=1}^{d} \langle v_i, U_j \rangle U_j$$

where $U^{[d]}$ is the transpose of the matrix whose d columns are $U_1, \ldots, U_d$. This is optimal in the sense that it minimises the mean values of the L_2 norms of the differences $v_i - v_{i,d}$.

Note F. Precision assessment without time stamps: Feng *et al.* and Cadanas *et al.*

In this section we explain how we calculate an upper bound on the variance of $P(T|g)$ from data where T is the TimeTeller estimated time and g is the gene expression vector or REV. This approach can also be used to get MAEs or MdAEs for this distribution. $P(T|g)$ gives the distribution of the TimeTeller estimate conditional on the gene expression state g. The methods currently used to quantify the precision of phase estimation algorithms focus on $P(T|t)$ where t is the sample time. This restricts such assessments to data that is time stamped. Moreover, for an individual sample the value $T - t$ is strongly biased by any chronotype for the individual, tissue or condition associated with the sample. For our human data we saw a range from -3.5h to 2.5h. Therefore, one can argue that it is more natural to instead try to assess the variance of $P(T|g)$ instead. We now describe in more detail how we do this.

We assume that we have a dataset and a determination of T for each data point. This might be the straightforward assessment of T by TimeTeller or it might be some adjusted value of T using TimeTeller such as the use of second peaks as done for the Feng *et al.* data. In addition, we will have the value of the REV g for each datapoint. We use the Feng *et al.* (*12*) data and that discussed in Cadenas *et al.* (*13*) as examples (SI Fig S16A).

We carry out PCA analysis as described in SI Note S6 where we take for the vectors v_j the REVs g in our dataset. We then project the REVs onto the first principal component $\mathbf{b}_1$ to get projected values $\tilde{g} = g \cdot \mathbf{b}_1$. From this we can produce a scatter plot as in Figs. 3I & SI Fig S16B where the projected value $\tilde{g}$ of g is plotted against the corresponding value of T. We then use kernel based smoothing or related methods to obtain a smooth curve $T = m(\tilde{g})$ giving the mean of the T values as a function of $\tilde{g}$ (Figs. 3I & SI Fig S16B) For the Feng *et al.* data the kernels used are normal with variance 0.5.

Then for each data point (with timing T_i and REV g_i) we calculate the quantity $T_i - m(\tilde{g}_i))$. These are the timing deviations. The resulting distributions are shown in (Figs. 3J & part C of SI Fig S16). We calculate the standard deviation (std) of these (overall std) and also check that it does not deviate much from the std found of we restrict to a subinterval of $\tilde{g}$ values with a reasonable number of data values in it (local std). This gives an estimate of the std of the distribution $P(T|\tilde{g})$ which is an upper bound of the std of $P(T|g)$.

For the Cadenas *et al.* data the analysis has been restricted to the data between $T = 10$ and $T = 20$.

For some datasets it will be necessary to generalise the approach and use more than one principal component in order to more accurately bound the variance.

Part II

Supplementary Figures

Fig A. Effect of time course normalisation

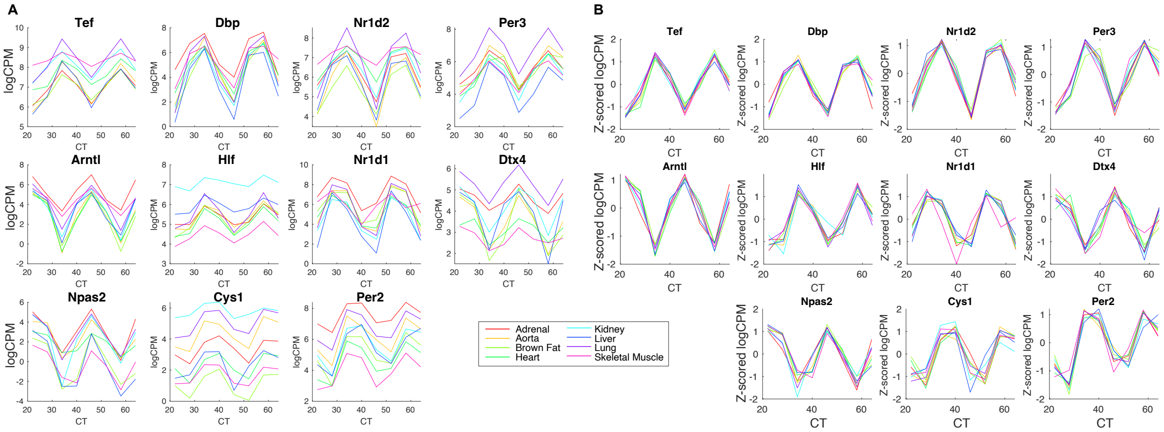

Time-dependent expression of the RNA-seq transcripts in the Zhang *et al.* RNA-seq training data. **A.** Before timecourse normalisation different tissues express the oscillatory genes at quite different amplitudes and magnitudes. **B.** After normalisation amplitudes and magnitudes are better aligned.

Fig B. Analysis of synchronicity and rhythmicity

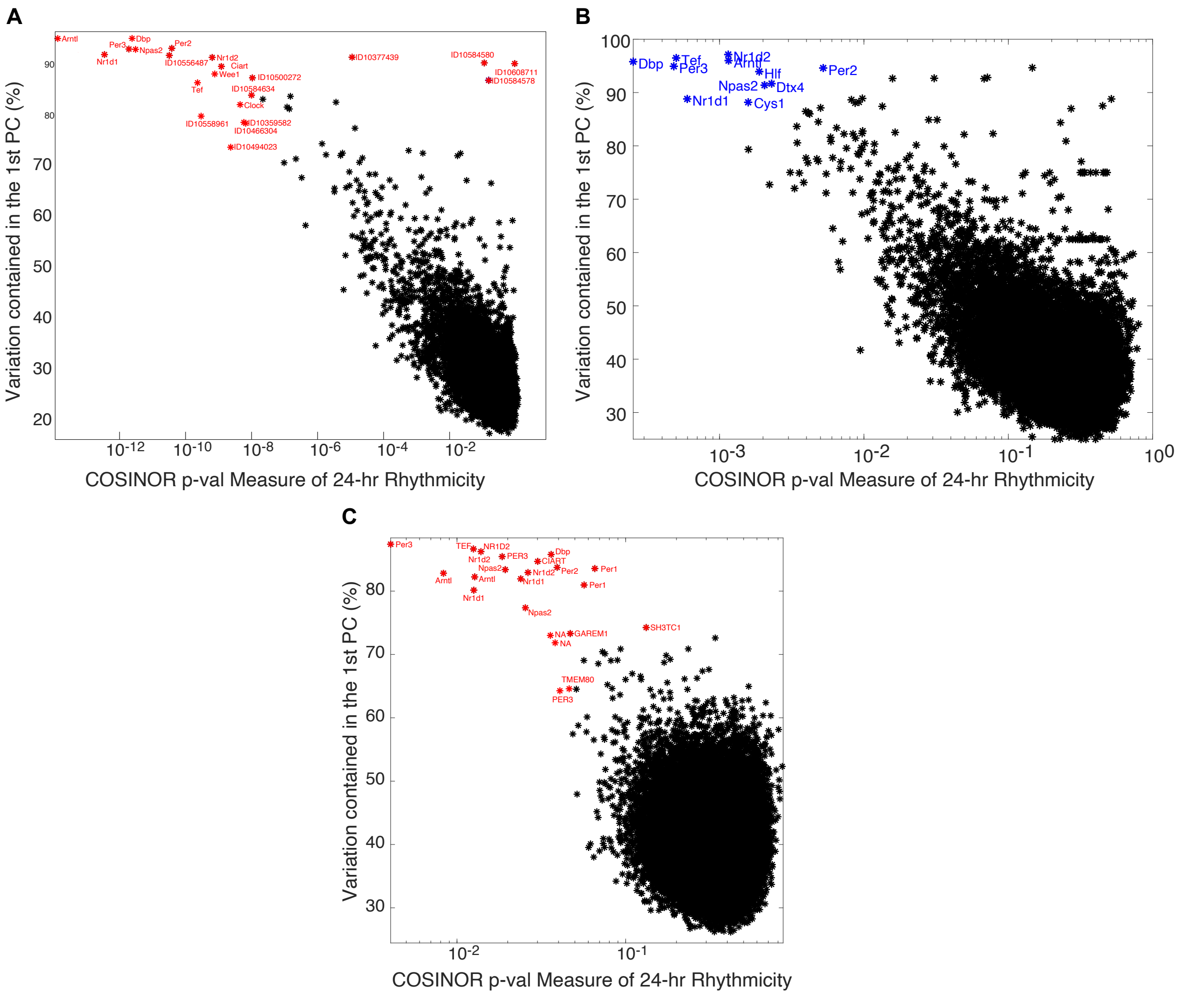

If we have q timepoints, for each gene g and each of the m organs or individuals we take the m q-dimensional timeseries vectors v_i and calculate their principal components $U_{g,i}$ and the associated singular values $\sigma_{g,i}$ as in SI Note S6. To characterise the relative strength of the first principal component we use $S_g^2 = \sigma_{g,1}^2 / \sum_k \sigma_{g,k}^2$ and this is our *synchronicity score*. These time-course normalised genes were ranked for goodness of 24 hr period cosine fit using the p-values from Cosinor analysis. One small change was made to the code, such that the p-value of the zero-amplitude f-test was calculated correctly using the fcdf function rather than the fpdf function. The p-values for the null hypothesis that the gene was not rhythmic were used to as our *periodicity score*. This was compared for consistency with analysis by JTK using the package at https://github.com/mfcovington/jtk-cycle. **A-C.** Scatter plot of the periodicity score against the synchronicity score. **A.** Zhang *et al.* (2014) microarray dataset. **B.** Zhang *et al.* (2014) RNA-seq dataset **C.** Bjarnason *et al.* microarray dataset.

Fig C. Hughes *et al.* data (*19*)

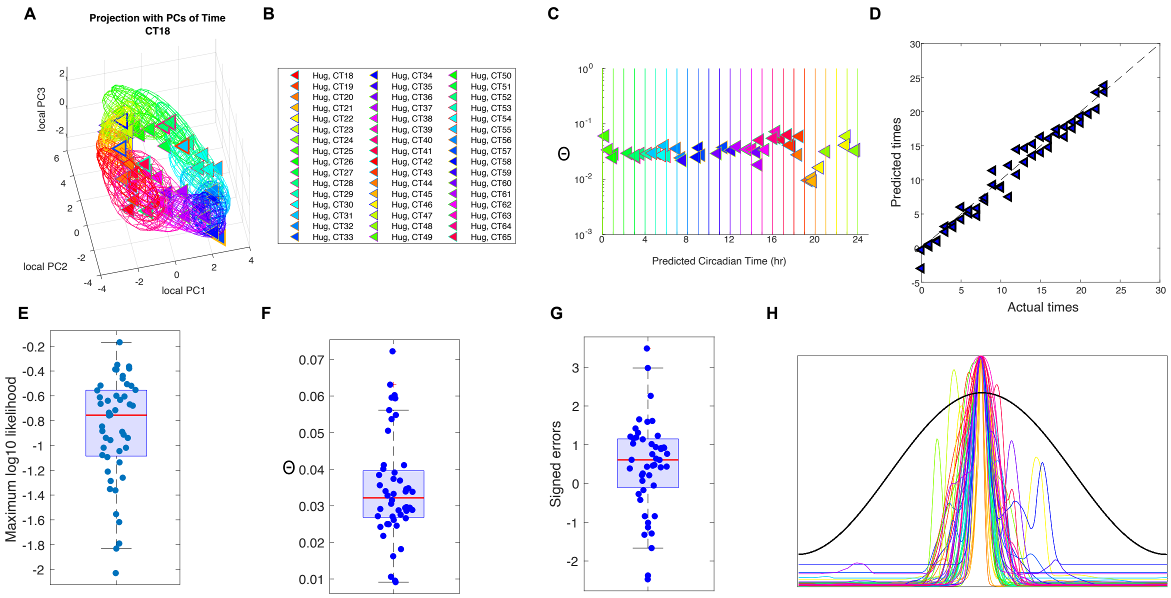

Analysis of Hughes *et al.* liver data (*19*) when $l_{\mathbf{thresh}} = -7$. Timecourse normalisation is used for both training and test data. **A.** Visualisation of the Hughes *et al.* liver data plotted against the curve given by the means of $P(g|t)$ for the Zhang *et al.* RNA-seq training data. **B.** Legend. Colors are consistent across all plots. **C.** Plot of the Θ values against the estimated time T for each test sample. The vertical lines show the true time with colours indicating the sampling time. **D.** Predicted T times plotted against actual times. **E.** Boxplot of the maximum likelihoods. **F.** Boxplot of the Θ values. **G.** Boxplot of the signed errors. **H.** Centred likelihood curves for each sample.

Fig D. Crossing platforms on Zhang *et al.* data

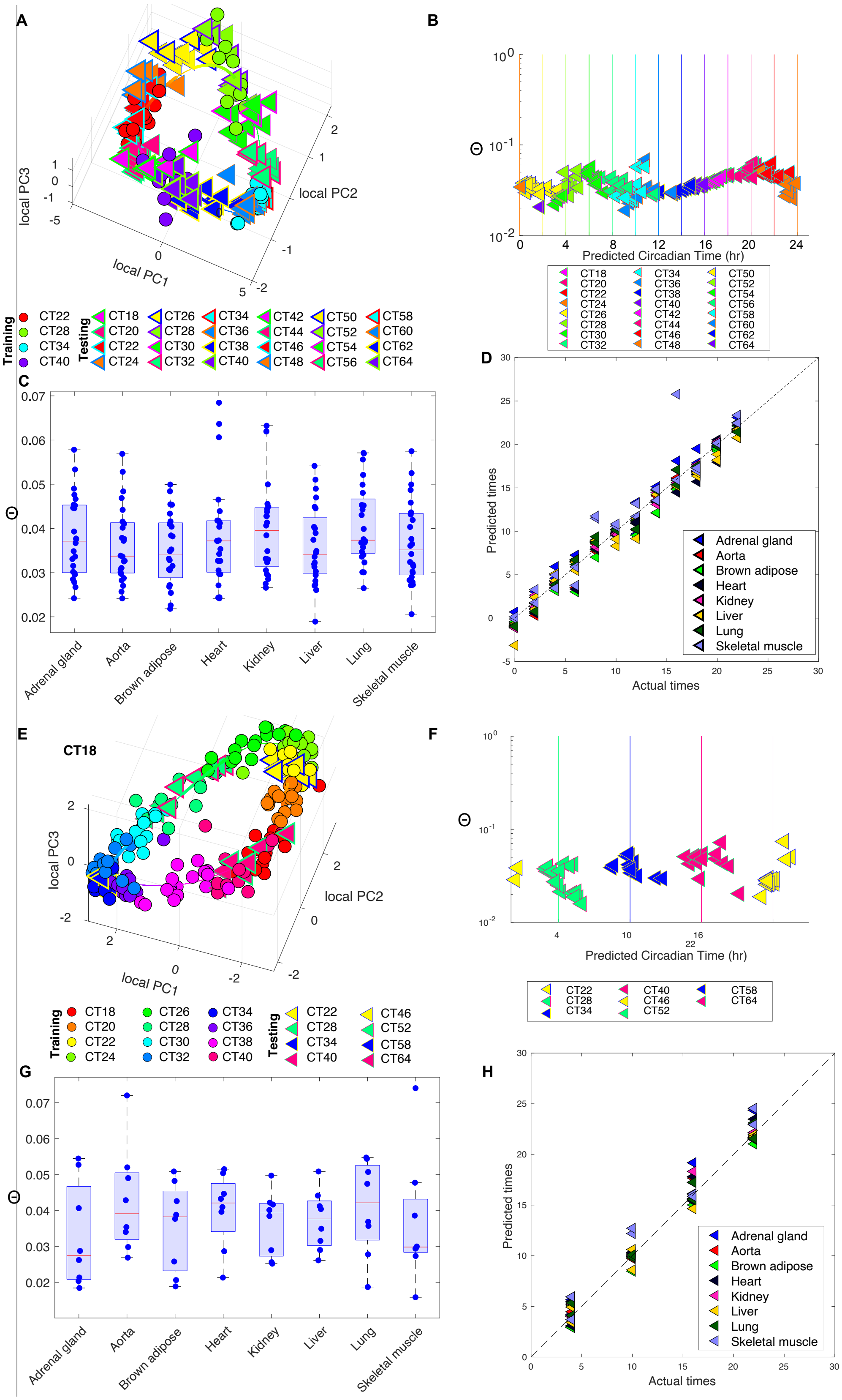

A-D. Results when testing Zhang *et al.* microarray data when trained on Zhang *et al.* RNA-seq data.
E-H. Results when testing Zhang *et al.* RNA-seq data when trained on Zhang *et al.* microarray data

Fig E. Local projections for human data.

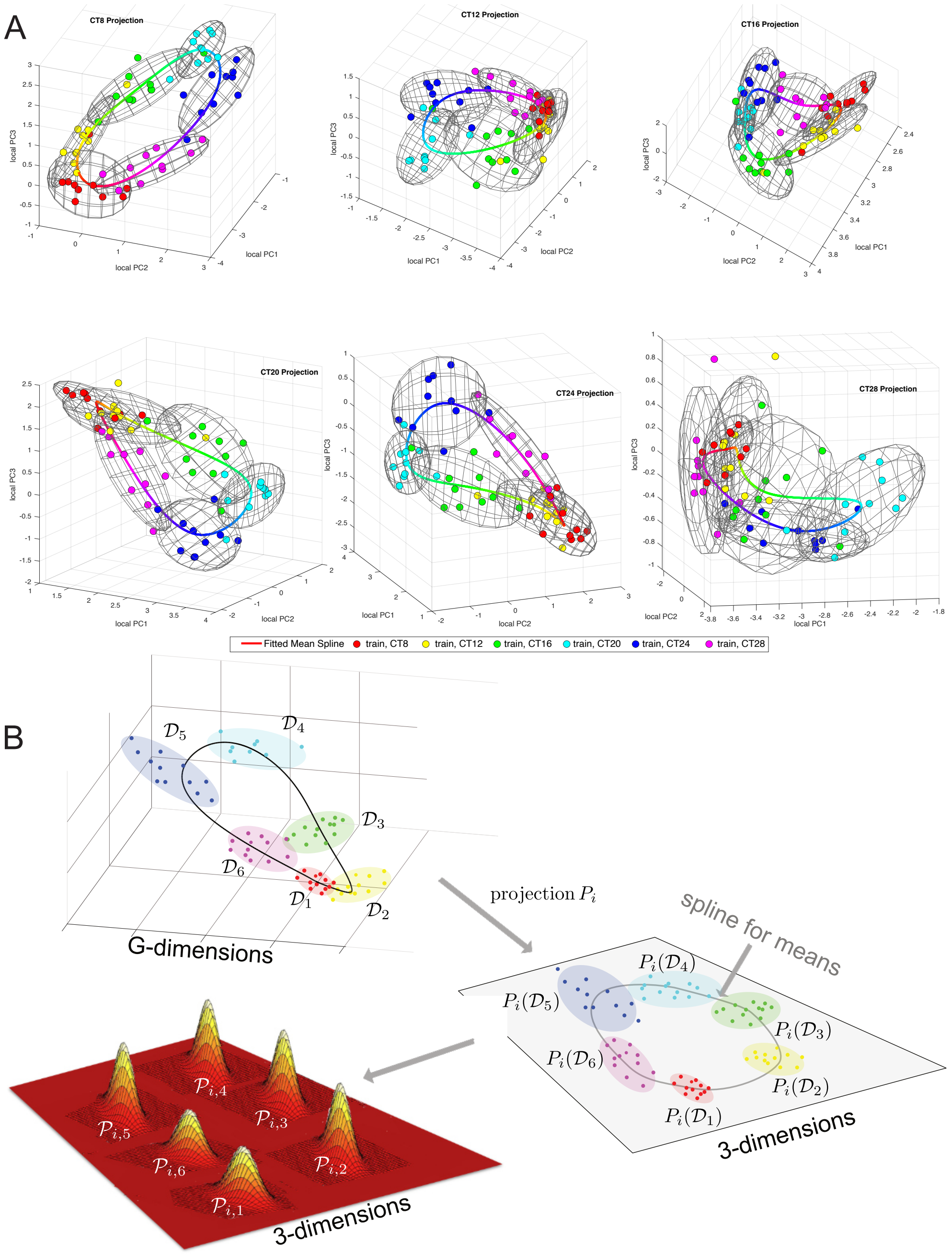

A. All 6 local projected spaces for human data. The normalised data is projected as described in Methods using the projections $U_{d,i}$ where $d = 3$ and i indexes the six times. Each time point is coloured according to its time. Splines through the means $\mu_i(t_j)$ of each set of 10 time data points show (distorted) elliptical shapes. Because of this shape we often have the situation where training data points at CTt are close to those of CT$(t+12)$ and this can give rise to two peaks in the likelihood curve $L_g(t)$ if the data point corresponding to g lies between the training data points at CTt and those of CT$(t + 12)$. **B. This schematic outlines the construction of the likelihoods $L_{g,i}(t)$.** For each i corresponding to the time t_i the set of normalised REVs $\mathcal{D}_i = \{g_{ij}, j = 1 \ldots, N_s\}$ are projected into $d = 3$ dimensions using the projection P_i to get $P_i(\mathcal{D}_j)$. A MVN distribution $\mathcal{P}_{i,j}$ is estimated for each $P_i(\mathcal{D}_j)$ and then these distributions are interpolated using splines to all times t of the day. The projections P_i for the Bjarnason *et al.* data and $d = 3$ is shown above in A.

Fig F. Variance explained by each PC.

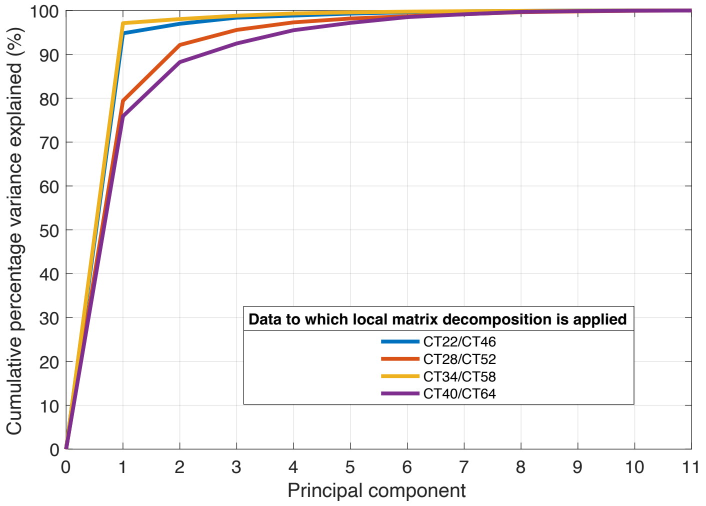

Cumulative percentage of variance explained by each of the principal components found by SVD for the Zhang *et al.* data. Data for day 1 and day 2 (e.g., CT22 and CT46) is combined to build a 24 hr model. 3 dimensions are sufficient to explain more than 90% of the variation in the dataset for each local projection. Such a situation follows when the eigenvalues of the covariance matrices of the $P(g|t_i)$ decay rapidly.

Fig G. Zhang *et al.* microarray data

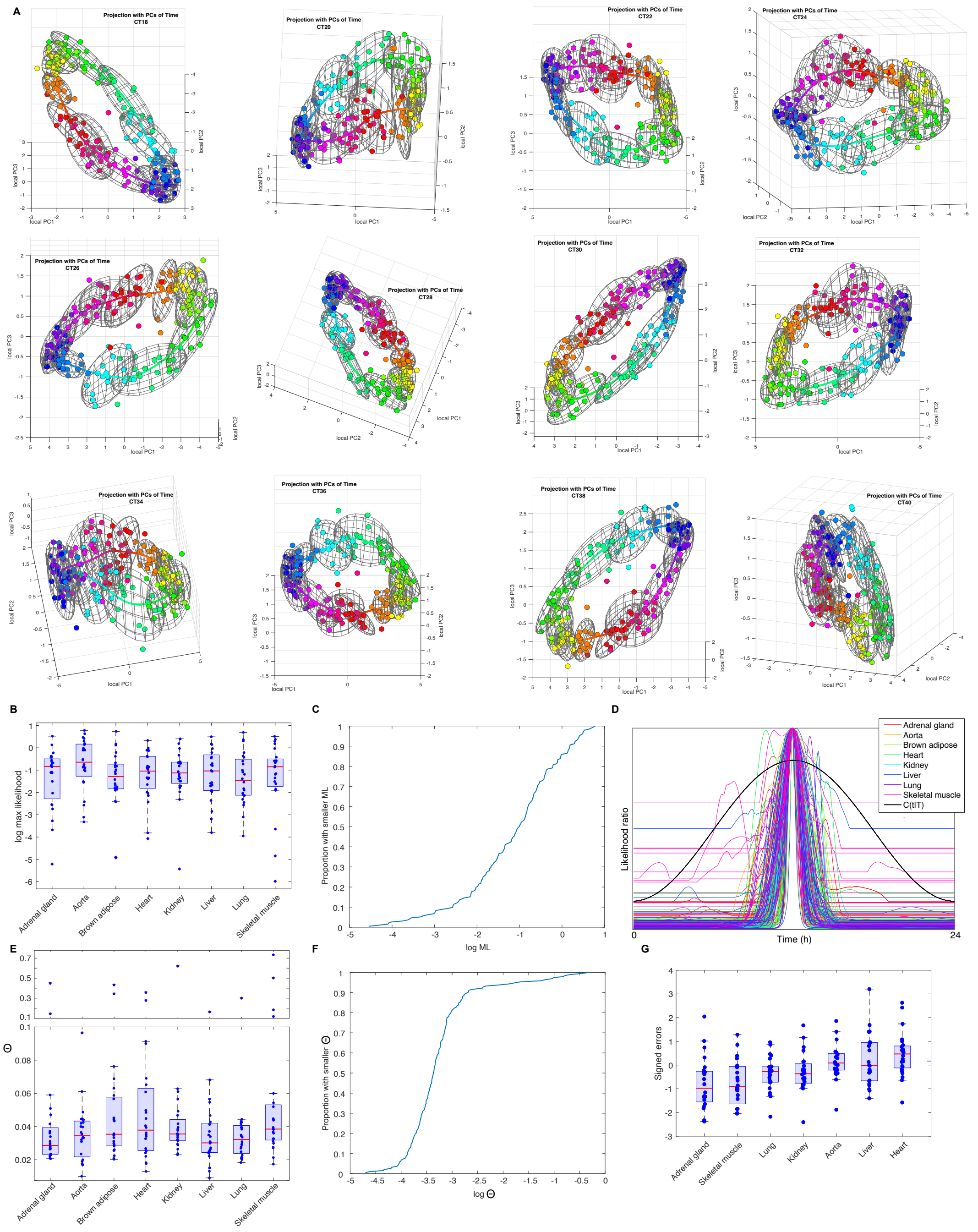

A. The twelve projections of the Zhang *et al.* microarray training data (one plot for each of the training times). **B-G.** Analysis of this data using a leave-one-tissue out cross-validation approach with $l_{\text{thresh}} = -5$. **B.** Box plots showing the log maximum likelihood values $\log \mathrm{ML}$ for each sample according to tissue. **C.** CDF of $\log \mathrm{ML}$ values found. Since only a few percent are less than -4 we take $l_{\text{thresh}} = -5$. $l_{\text{thresh}} = -4$ would also have been appropriate. **D.** Centered likelihood ratio curves (CLRFs). Note that although there are a number of samples with flat regions intersecting $C(t|T)$, by B, these are only a small proportion of all samples. About 15% of the CLRFs have two peaks. **E.** Box plots showing the Θ values for each tissue. **F.** CDF of $\log \Theta$ values found. Note that only about 5% have $\log \Theta > -2$. **G.** Signed timing errors for the different tissues. The means give the timing displacements.

Fig H.   Analysis of Kinouchi *et al.* liver data.

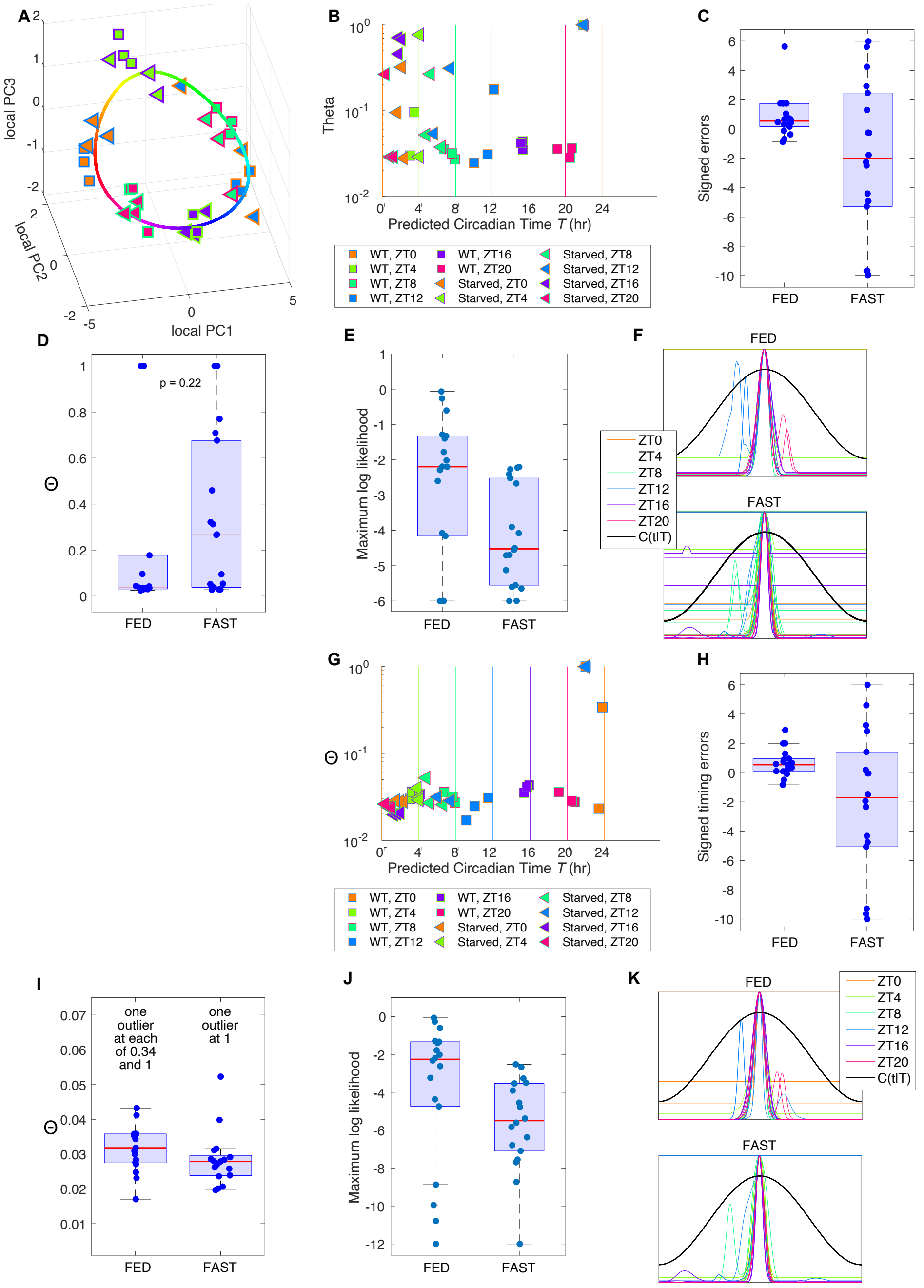

Analysis of Kinouchi *et al.* liver data. **A.** Visualisation of the Kinouchi *et al.* liver data plotted against the curve given by the means of $P(g|t)$ for the Zhang *et al.* RNA-seq training data. **B-E.** These use $l_{\text{thresh}} = -6$. **B.** Plot of the Θ values against the estimated time T for each test sample. The vertical lines show the true time with colours indicating the sampling time. **C.** Boxplots of the signed errors showing that the absolute errors of the errors for FAST data are significantly higher than those for FED data. The mean values for FED and FAST are respectively 1.23h and 4.70h and the medians 0.78h and 4.46h. The difference is significant at the $p < 0.0008$ level. **C & D.** Boxplots of the Θ and maximum likelihood values. **E.** The likelihood curves for the FED and FAST samples. These have been been translated in time so that its highest peak is at 12noon as this makes comparison of the shapes easier. **F.** Centred LRFs for the FED and FAST mice. **G-K.** As B-E but these use $l_{\text{thresh}} = -12$.

Fig I. Bjarnason *et al.* data

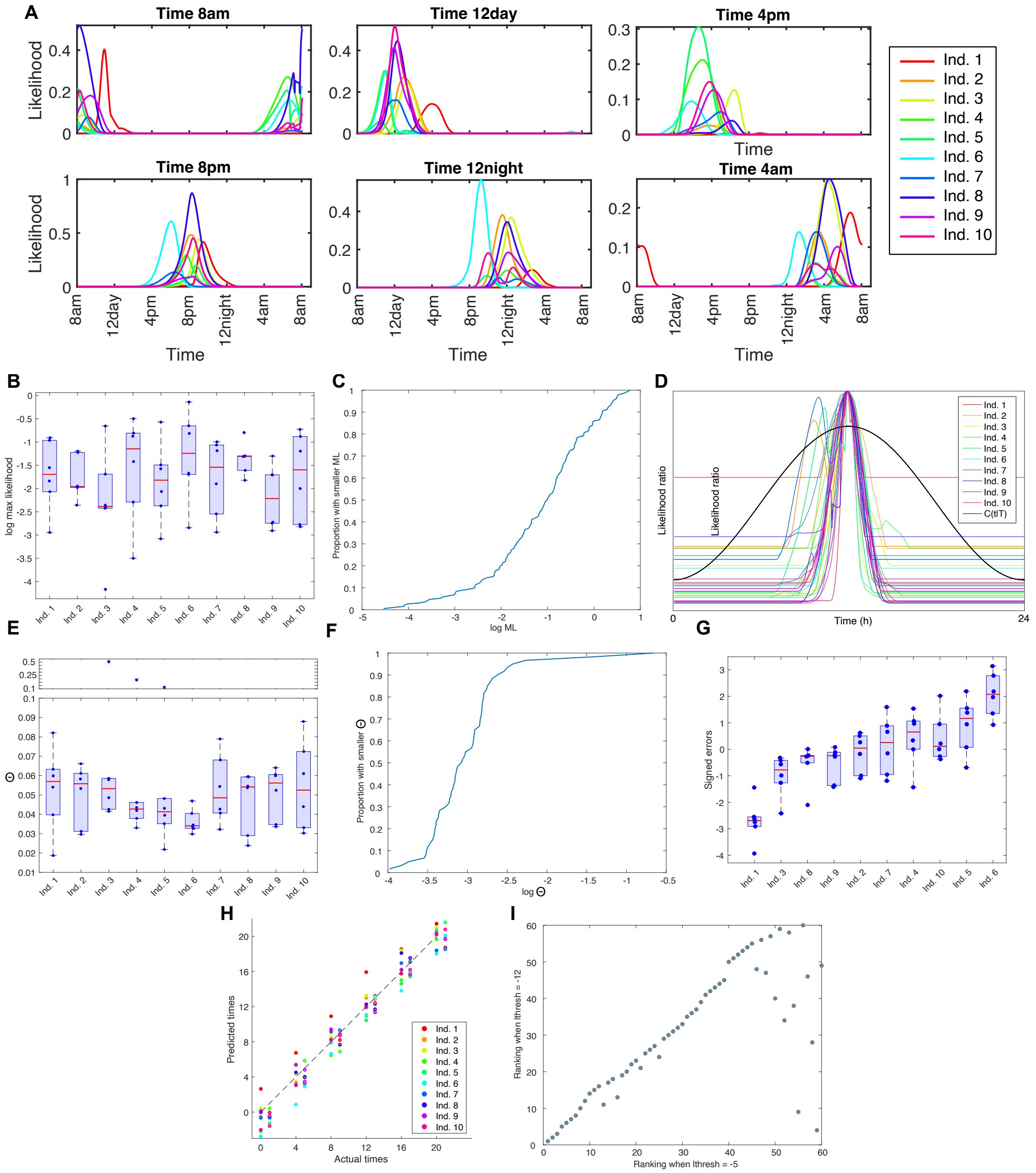

A. The likelihood curves for the leave-one-out analysis of the Bjarnason *et al.* data. The sample time is shown above each subplot. **B-I.** Leave-one-individual-out analysis of Bjarnason *et al.* training data. Intergene normalisation is used with $l_{\text{thresh}} = -5$. **B.** Boxplots showing the distribution of the log maximum likelihood values $\log \mathrm{ML}$ by individual. **C.** CDF of the $\log \mathrm{ML}$ values. **D.** Centred likelihood ratio functions for all individuals at all timepoints. **E.** Boxplots showing the distribution of Θ values by individual. **F.** CDF of the Θ values. **G.** Boxplots showing the distribution of the signed errors for each individual. The means increase from right to left. **H.** Analysis of the timing for the Bjarnason *et al.* human microarray data with each data point assigned the color corresponding to the individual. For each time the points over the time are for uncorrected timing and the points moved slightly to the right show the corrected timings (i.e. the predicted timing when corrected by the timing displacement for the individual). **I.** An analysis of how the Θ stratification changes as we lower l_{thresh} from -5 to -12. The scatter plot shows the change in the Θ stratification of samples when l_{thresh} is changed from -5 to -12. While 14 points change their ranking, for almost all the change preserves whether they are high or low. Only two samples change from high to low. The reason we generally choose $l_{\text{thresh}} = -5$ here is because (i) higher values have too many samples in the training data with flat regions significantly intersecting $C(t|T)$ and (ii) lower values are too far below the minimum ML of the test data.

Fig J. Θ values for Mure *et al.* data

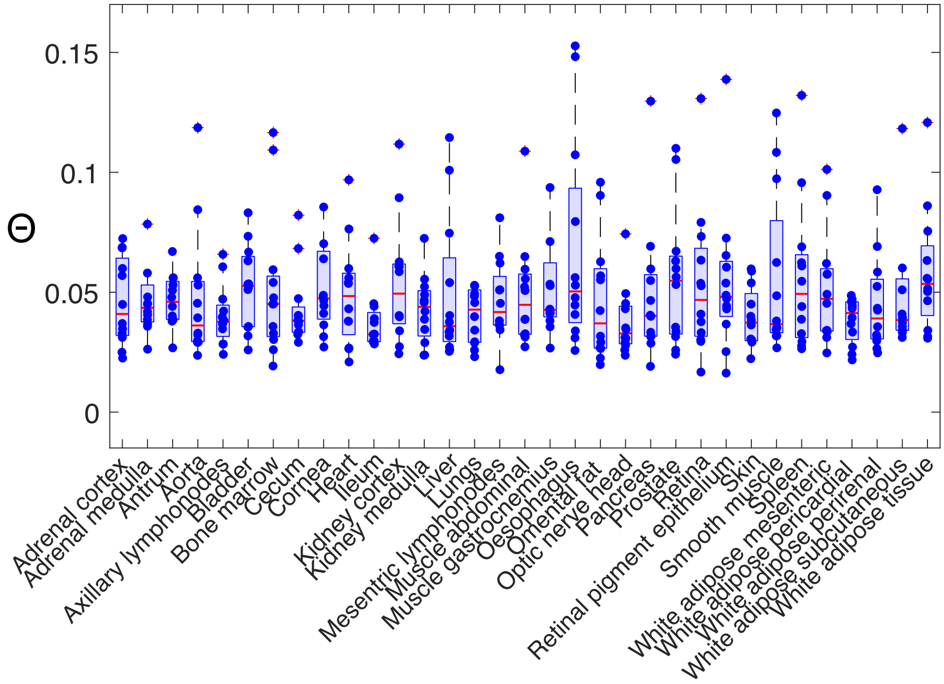

This shows the Θ values for the 33 tissues studied. The following tissues have 1 high outlier which is not shown: cornia, adrenal med., white adipose, lungs, muscle gast. . Only the central 18 tissues were used for training and $l_{\text{thresh}} = -12$.

Fig K. Analysis of *Arntl* and *Cr1/Cry2* knockouts.

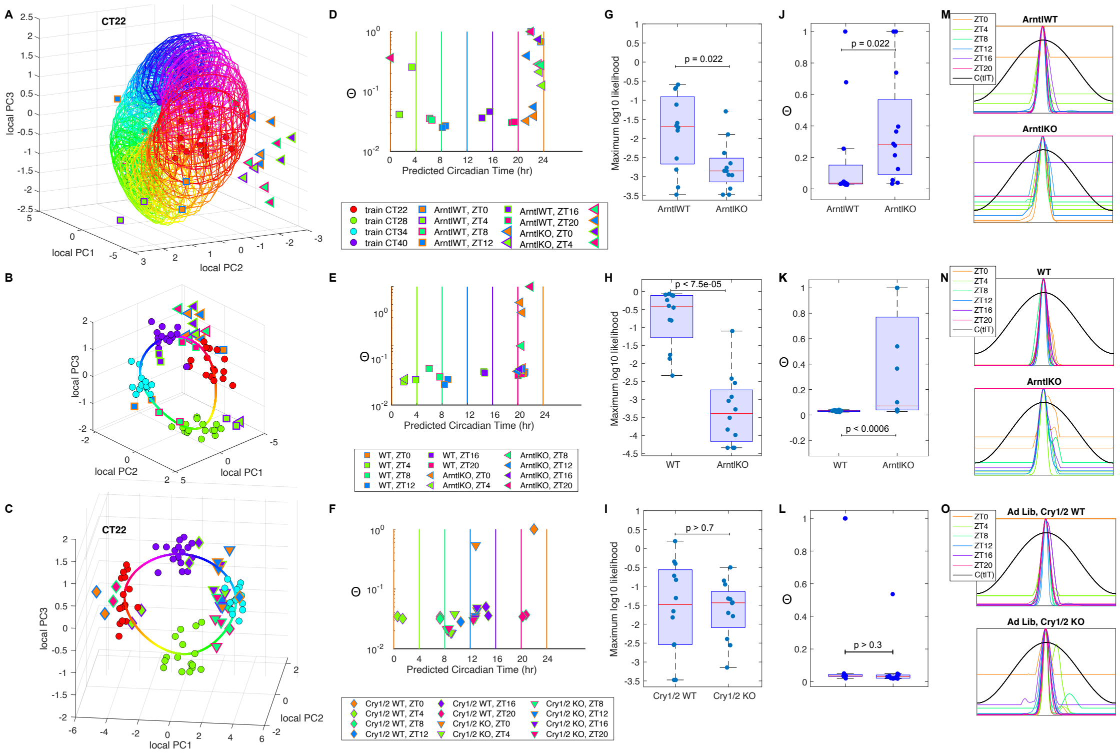

Visualisation and quantitative analysis of *Arntl* and *Cr1/Cry2* knockouts. Row 1: Weger *et al.* *Arntl*KO (*3*); row 2: Yeung *et al.* *Arntl*KO (*20*); row 3: Weger *et al.* *Cr1/Cry2*KO (*3*). All shown p-values are from the Wilcoxon Rank-Sum test. **A, B & C.** The visualisation of the data for each dataset compared to the training data. In each case the clustering of the KO data about a particular timepoint is evident. **D, E & F.** Plots of the Θ value against the estimated time T. The vertical lines show the true time with colours indicating the sampling time as shown in the various legends. These plots show the clustering of the KO clocks around a specific time of day.

Fig L. Boyle *et al.* data & Feng *et al.* data.

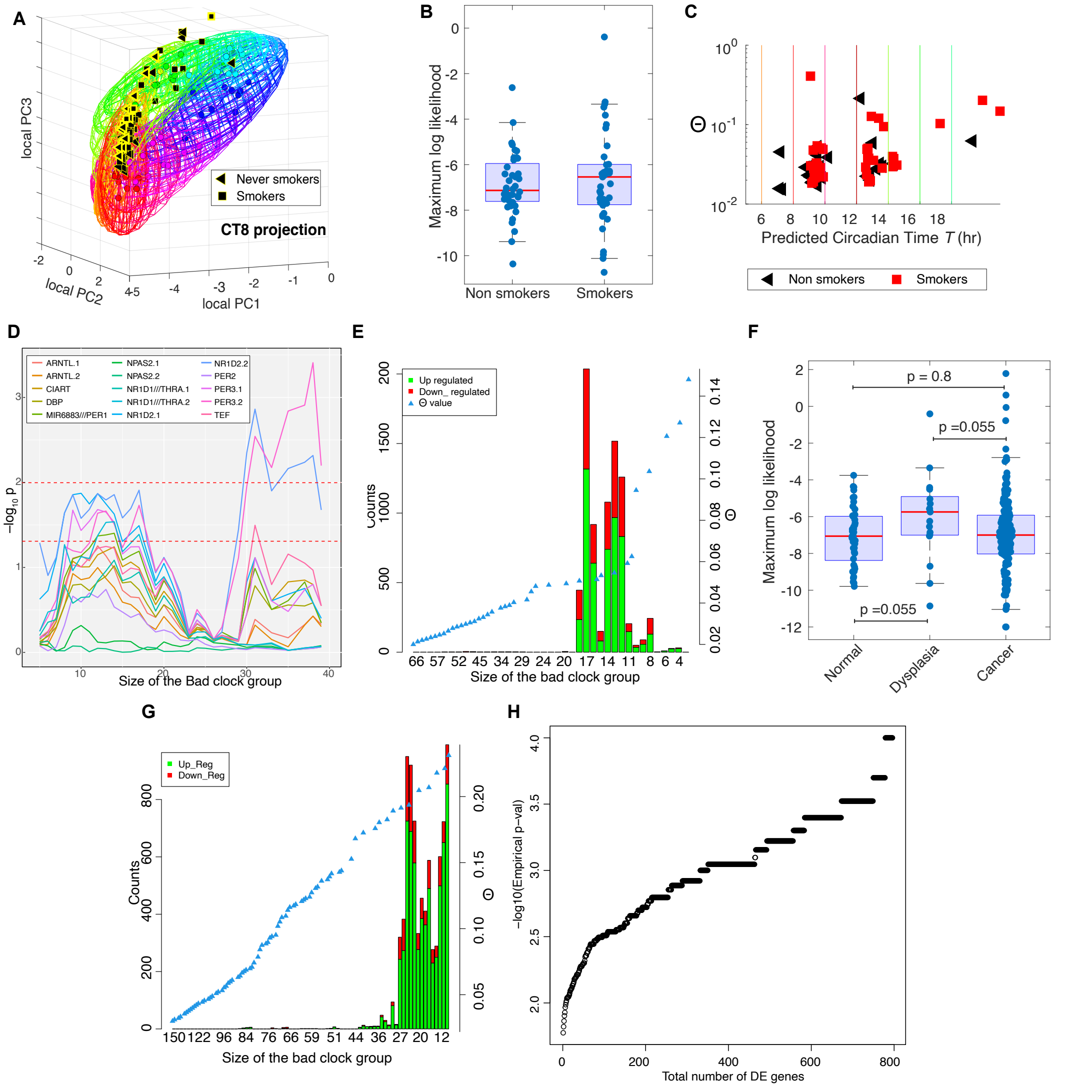

Analysis of Boyle *et al.* smoking data (*11*). A. Visualisation of the Boyle *et al.* data showing how this data sits in the Bjarnason *et al.* microarray training data. **B-H.** This uses intergene normalisation and $l_{\text{thresh}} = -12$. **B.** ML values for smokers and non-smokers. **C.** Scatter plot of Θ values against estimated timing T. This uses $l_{\text{thresh}} = -12$. **D.** The adjusted p-values for clock genes which are differentially expressed between those n individuals with the worse clocks (according to the Θ stratification) and those with better clocks. The p-values were calculated using the limma package (v3.48.3) and have been adjusted to account for the multiple testing with the BH method used for adjustment. Some of the genes had two probes which are distinguished by a point followed by either 1 or 2. The broken red lines show where $p = 0.05$ and $p = 0.01$. **E.** The number of statistically significant differentially expressed genes as a function of n, the size of the bad clock group. The threshold for differential expression is $p < 0.05$ for the adjusted p-value (limma using BH). The blue triangles indicate the corresponding Θ values. **F-H. Analysis of Feng *et al.* data (*12*).** This uses intergene normalisation and $l_{\text{thresh}} = -12$. **F.** ML values for the normal, dysplasic and cancer data. **G.** As B but for the Feng *et al.* data. **H.** Empirical probability of observing m or more differentially expressed genes (DEGs) 10,000 simulations were run in which the number of DEGs were calculated after the bad clock group size was randomised to between 15 and 40 and individuals were randomly allocated to the bad and good clock groups. The empirical distribution plot shows the results of observing m or more DE genes as per the distribution obtained. Plotted on -log10 scale.

Fig M. PCP plots for Bjarnason *et al.* data

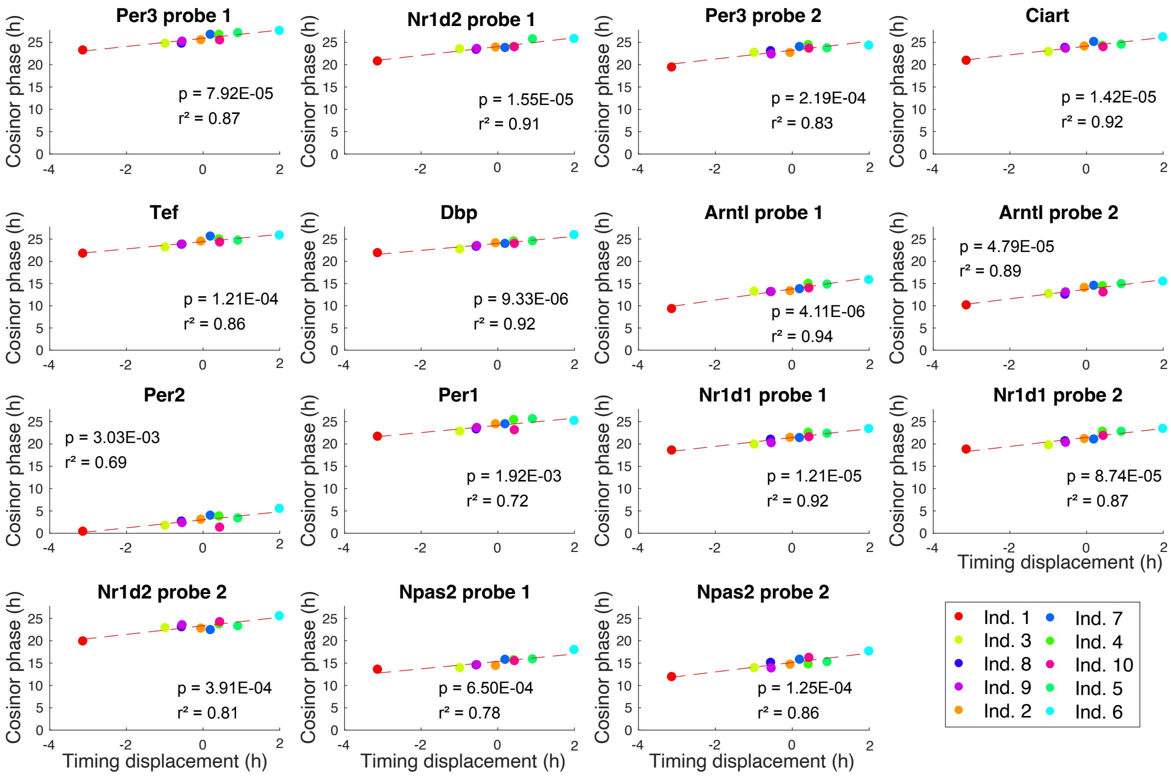

This shows the strong linear relationship between the gene phases and the timing deviation in the Bjarnason *et al.* data. Each point corresponds to an individual. The regression was carried out using Matlab's fit function and Cosinor (*?*) was used to estimate the gene phases from the time series of each individual. The p-values test the hypothesis the the slope of the line is non-zero and are given by the F-test using the Matlab functions coefTest and fitlm. The r^2 values measure of the proportion of total variation of gene phase explained by the linear model. They indicate the extent to which the linear model (corresponding to a simple phase change) explains the data with the values very close to 1 almost completely explaining it.

Fig N. Ageing genes identified in Acosta-Rodríguez *et al.*

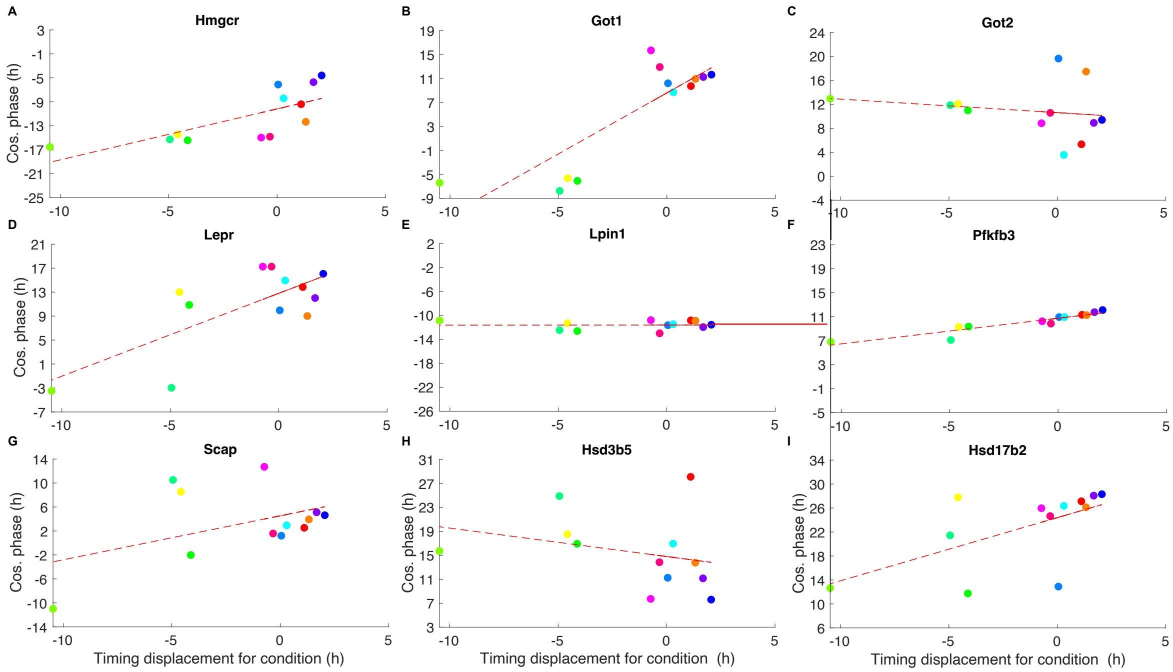

J

	Hmgcr	Got1	Got2	Lepr	Lpin1	Pfkfb3	Scap	Hsd17b2	Hsd3b5
P-value	7.58E-03	1.58E-03	4.29E-01	8.85E-02	9.84E-01	7.33E-06	2.57E-02	3.60E-01	3.69E-01
R-squared	0.53	0.65	0.06	0.26	0.00	0.88	0.41	0.08	0.08

K

AL 6 mo. · AL 19 mo. · CR day 2h 6 mo. · CR day 2h 19 mo. · CR day 12h 6 mo. · CR day 12h 19 mo. · CR night 2h 6 mo. · CR night 2h 19 mo. · CR night 12h 6 mo. · CR night 12h 19 mo. · CR spread 6 mo. · CR spread 19 mo.

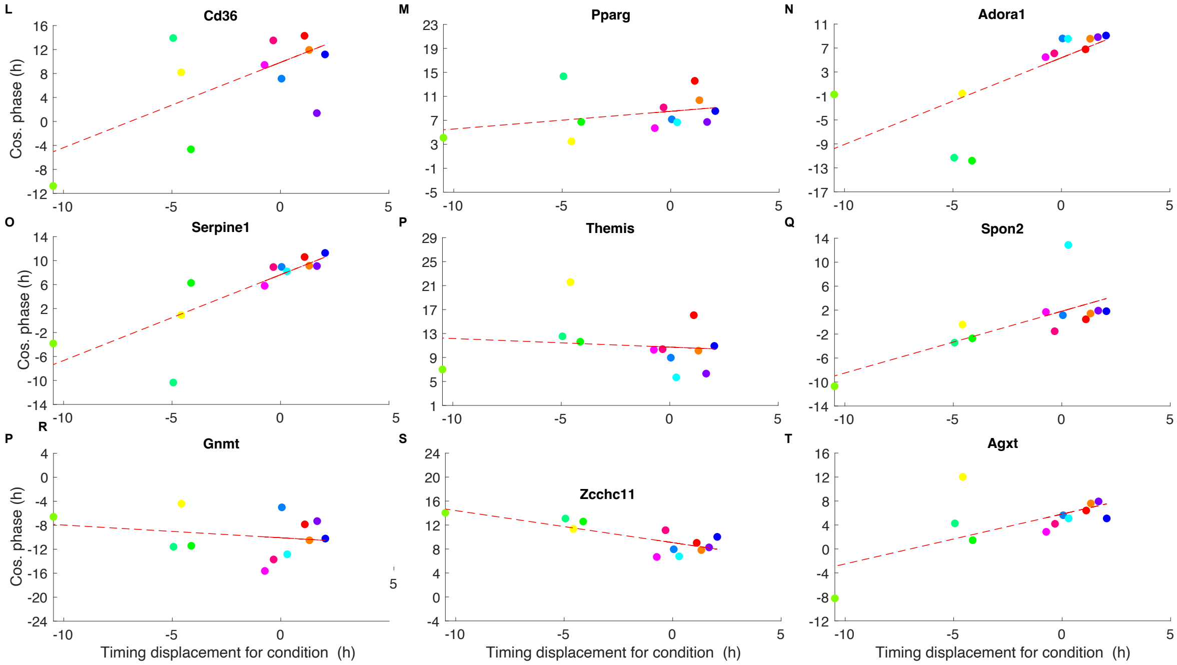

U

	Cd36	Pparg	Adora1	Serpine1	Themis	Spon2	Zcchc11	Gnmt	Agxt
p-value	3.51E-01	2.99E-01	9.12E-04	1.28E-03	7.06E-01	3.38E-02	2.08E-03	4.83E-01	7.76E-02
r-squared	0.09	0.11	0.68	0.66	0.01	0.38	0.63	0.05	0.28

V

AL 6 mo. · AL 19 mo. · CR day 2h 6 mo. · CR day 2h 19 mo. · CR day 12h 6 mo. · CR day 12h 19 mo. · CR night 2h 6 mo. · CR night 2h 19 mo. · CR night 12h 6 mo. · CR night 12h 19 mo. · CR spread 6 mo. · CR spread 19 mo.

PCPs for a subset of the geing genes from (*7*). The Zhang *et al.* data was used for training, timecourse-matched normalisation was used for test data and l_{thresh} = -8.

Fig O. Choice of l_{thresh}.

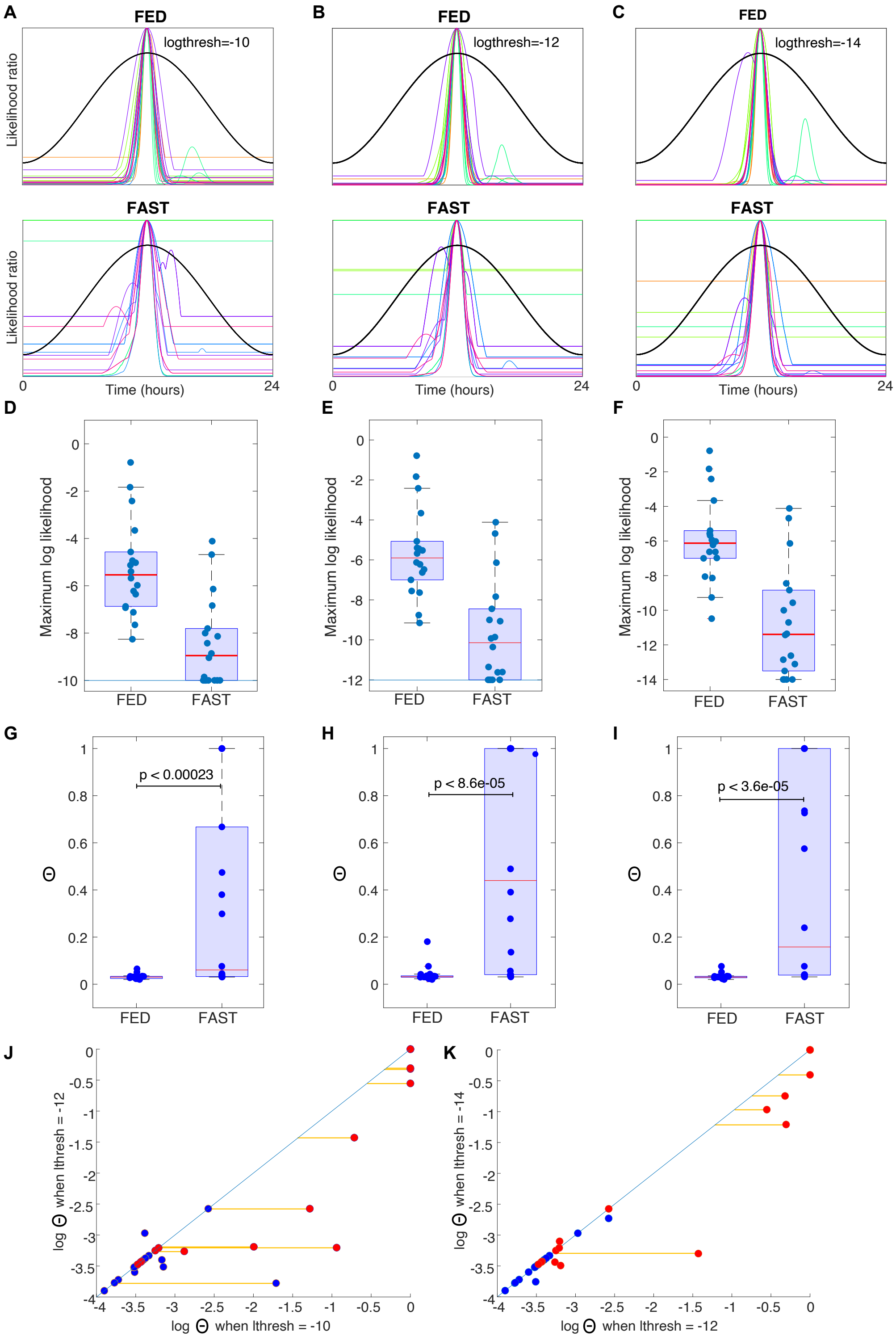

Choosing l_{thresh}. The data shown is for the Kinouchi *et al.* skeletal muscle data. **A-C.** Centre LRFs for $l_{\text{thresh}} = -10, -12$ and -14. **D-I.** Box plots of the MLs and Θs for the three values of l_{thresh}. In D-F the blue horizontal line shows the value of the threshold $\exp(l_{\text{thresh}})$. p-values shown are from the Wilcoxon rank sum test (Matlab function ranksum). **J.** Scatter plot showing how Θ and the ordering of Θs change when l_{thresh} is decreased. Blue points FED, red points FAST. Orange lines guide the eye when checking whether a sample changes its position in the Θ stratification. Just one point does this in K.

Fig P. Precision assessment without time stamps: Cadanas *et al.*

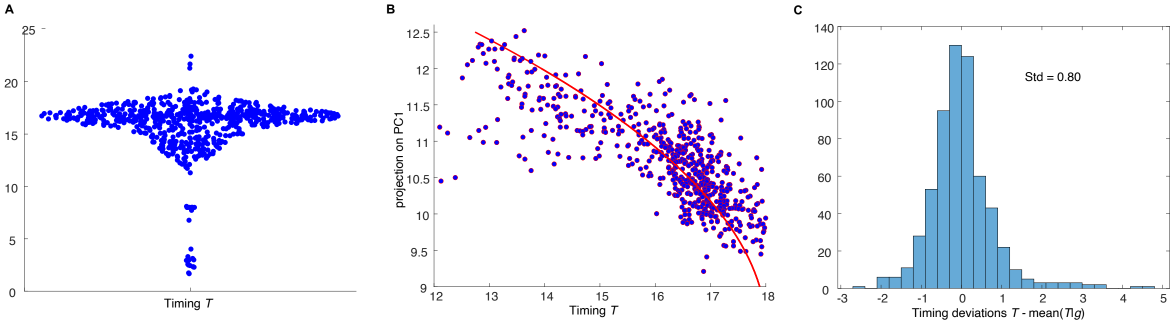

Swarm chart showing the TimeTeller timings T of the Cadenas *et al.* data. **B.** A scatter plot of the projection $\tilde{g}$ of the data REVs g onto the first PC (vertical axis) against the TimeTeller timing T. The smooth red curve approximates the means of $P(T|\tilde{g})$. **C.** The distribution of the deviations i.e. the horizontal difference between each data point $(T, \tilde{g})$ and the mean of $P(T|\tilde{g})$. The standard deviation of this distribution is shown.

Part III

Supplementary Tables

Table A. Use of normalisations.

Use of normalisation			
Dataset	Training normalisation	Test normalisation	Figure
Acosta-Rodeguez *et al.*	timecourse	timecourse-matched	4
Acosta-Rodeguez *et al.*	timecourse	timecourse	not shown
Bjarnason *et al.*	intergene	intergene	1,2,4
Boyle *et al.*	intergene	intergene	3
Fang *et al.*	intergene	intergene	1,2
Feng *et al.*	intergene	intergene	3
Hughes *et al.*	timecourse	timecourse	SI Fig S2
Kinouchi *et al.* skel. mus.	timecourse	timecourse-matched	1,2
Kinouchi *et al.* liver	timecourse	timecourse-matched	SI Fig S8
Koronowski *et al. et al.*	timecourse	timecourse-matched	2
Le Martelot *et al.*	intergene	intergene	not shown
Mure *et al.*	timecourse	timecourse-matched	4
Weger *et al.*	timecourse	timecourse-matched	SI Fig S9
Yeung *et al.*	timecourse	timecourse-matched	SI Fig S9
Zhang *et al.* microarray	intergene	intergene	1, 2
Zhang *et al.* microarray	timecourse	timecourse-matched	1, 2, 3, 4
Zhang *et al.* RNA-seq	timecourse then intergene		1
Zhang *et al.* RNA-seq	timecourse	timecourse-matched	2,4, SI Fig S8, SI Fig S9

This shows where the different normalisations are used in the data presented. Where test data normalisation is given for a training dataset this refers to how the test data comes from a leave one out analysis.

Table B. Table Timing comparison for Zhang *et al.* microarray data

	Adrenal	Aorta	Brown adipose	Heart	Kidney	Liver	Lung	Skeletal muscle
ZeitZeiger	1.08	0.53	0.60	0.68	0.66	0.66	0.62	1.50
TimeTeller	1.14	0.50	0.83	0.76	0.67	0.85	0.61	1.75
TimeTeller corrected	0.88	0.50	0.58	0.68	0.57	0.88	0.53	1.55

This compares the mean absolute timing errors for the eight tissues used in the microarray training data from Zhang *et al.* . The top row are for ZeiZeiger and are extracted from Fig. S3 of (*21*). The 2nd and 3rd row are for TimeTeller using timecourse normalisation and the timing errors in the 3rd row have been corrected using the timing deviations for the tissues.

Table C. Mure *et al.* data

Gene	Arntl	Ciart	Nr1d2	Nr1d1	Tef	Per3	Npas2	Dbp	Per1	RorC	Cry1	Per2	Cry2
P-value	4.4E-7	3.0E-8	3.4E-3	3.0E-5	3.0E-4	1.7E-3	4.6E-02	5.2E-7	1.9E-3	6.1E-1	1.9E-2	1.9E-4	5.2E-1
R^2	0.57	0.63	0.25	0.43	0.35	0.28	0.12	0.56	0.27	0.01	0.17	0.37	0.01

Mure *et al.* data The p-value and r^2 values for the linear regression of the gene phase of each gene shown in the plots against the timing displacements of the the 33 tissues for the Mure *et al.* data. TimeTeller has been trained on the central tissues only and l_{thresh} = -12.

Table D. Significance of Θ differences between conditions of Acosta-Rodríguez *et al.* data (*7*)

	AL6m	AL19m	CRday12h19m	CRnight2h6m	CRnight2h19m	CRnight12h6m	CRnight12h19m	CRspread6m	CRspread19m
AL6m				1.30E-02	3.82E-02				
AL19m				2.20E-03	7.13E-03				
CRday2h6m									
CRday2h19m									
CRday12h6m									
CRday12h19m				4.89E-02					
CRnight2h6m	1.30E-02	2.20E-03	4.89E-02			4.66E-02	4.89E-02	1.22E-02	5.91E-03
CRnight2h19m	3.82E-02	7.13E-03							2.15E-02
CRnight12h6m				4.66E-02					
CRnight12h19m				4.89E-02					
CRspread6m				1.22E-02					
CRspread19m			5.91E-03	2.15E-02					

This table shows p-values for the Wilcoxon rank sum test (Matlab function ranksum) testing the null hypothesis that the Θ values for the indicated pair of conditions arise from distributions with equal medians. Only those with $p < 0.05$ are shown.

Bibliography

1. R. Zhang, N. F. Lahens, H. I. Ballance, M. E. Hughes, and J. B. Hogenesch, "A circadian gene expression atlas in mammals: Implications for biology and medicine," *Proceedings of the National Academy of Sciences*, pp. 2–7, oct 2014.

2. K. Kinouchi, C. Magnan, N. Ceglia, Y. Liu, M. Cervantes, N. Pastore, T. Huynh, A. Ballabio, P. Baldi, S. Masri, *et al.*, "Fasting imparts a switch to alternative daily pathways in liver and muscle," *Cell reports*, vol. 25, no. 12, pp. 3299–3314, 2018.

3. B. D. Weger, C. Gobet, F. P. David, F. Atger, E. Martin, N. E. Phillips, A. Charpagne, M. Weger, F. Naef, and F. Gachon, "Systematic analysis of differential rhythmic liver gene expression mediated by the circadian clock and feeding rhythms," *Proceedings of the National Academy of Sciences*, vol. 118, no. 3, 2021.

4. B. Fang, L. J. Everett, J. Jager, E. Briggs, S. M. Armour, D. Feng, A. Roy, Z. Gerhart-Hines, Z. Sun, and M. A. Lazar, "Circadian enhancers coordinate multiple phases of rhythmic gene transcription in vivo," *Cell*, vol. 159, no. 5, pp. 1140–1152, 2014.

5. J. L. Barclay, J. Husse, B. Bode, N. Naujokat, J. Meyer-Kovac, S. M. Schmid, H. Lehnert, and H. Oster, "Circadian desynchrony promotes metabolic disruption in a mouse model of shiftwork," *PLoS ONE*, vol. 7, no. 5, 2012.

6. G. Le Martelot, D. Canella, L. Symul, E. Migliavacca, F. Gilardi, R. Liechti, O. Martin, K. Harshman, M. Delorenzi, B. Desvergne, W. Herr, B. Deplancke, U. Schibler, J. Rougemont, N. Guex, N. Hernandez, and F. Naef, "Genome-Wide RNA Polymerase II Profiles and RNA Accumulation Reveal Kinetics of Transcription and Associated Epigenetic Changes During Diurnal Cycles," *PLoS Biology*, vol. 10, no. 11, 2012.

7. V. Acosta-Rodriguez, F. Rijo-Ferreira, M. Izumo, P. Xu, M. Wight-Carter, C. Green, and J. Takahashi, "Circadian alignment of early onset caloric restriction promotes longevity in male c57bl/6j mice.," *Science*, vol. 376, no. 6598, pp. 1192–1202, 2022.

8. K. B. Koronowski, K. Kinouchi, P.-S. Welz, J. G. Smith, V. M. Zinna, J. Shi, M. Samad, S. Chen, C. N. Magnan, J. M. Kinchen, *et al.*, "Defining the independence of the liver circadian clock," *Cell*, vol. 177, no. 6, pp. 1448–1462, 2019.

9. L. S. Mure, H. D. Le, G. Benegiamo, M. W. Chang, L. Rios, N. Jillani, M. Ngotho, T. Kariuki, O. Dkhissi-Benyahya, H. M. Cooper, *et al.*, "Diurnal transcriptome atlas of a primate across major neural and peripheral tissues," *Science*, vol. 359, no. 6381, p. eaao0318, 2018.

10. G. Bjarnason, A. Seth, Z. Wang, N. Blanas, M. Straume, and T. Martino, "Diurnal rhythms (dr) in gene expression in human oral mucosa: Implications for gender differences in toxicity, response and survival and optimal timing of targeted therapy (rx)," *Journal of Clinical Oncology*, vol. 25, no. 18_suppl, pp. 2507–2507, 2007.

11. J. O. Boyle, Z. H. Gümüş, A. Kacker, V. L. Choksi, J. M. Bocker, X. K. Zhou, R. K. Yantiss, D. B. Hughes, B. Du, B. L. Judson, K. Subbaramaiah, and A. J. Dannenberg, "Effects of cigarette smoke on the human oral mucosal transcriptome," *Cancer Prevention Research*, vol. 3, no. 3, pp. 266–278, 2010.

12. L. Feng, J. R. Houck, P. Lohavanichbutr, and C. Chen, "Transcriptome analysis reveals differentially expressed lncRNAs between oral squamous cell carcinoma and healthy oral mucosa.," *Oncotarget*, vol. 8, no. 19, pp. 31521–31531, 2017.

13. C. Cadenas, L. van de Sandt, K. Edlund, M. Lohr, B. Hellwig, R. Marchan, M. Schmidt, J. Rahnenführer, H. Oster, and J. G. Hengstler, "Loss of circadian clock gene expression is associated with tumor progression in breast cancer.," *Cell cycle (Georgetown, Tex.)*, vol. 13, pp. 3282–91, oct 2014.

14. V. Nygaard, E. A. Rødland, and E. Hovig, "Methods that remove batch effects while retaining group differences may lead to exaggerated confidence in downstream analyses," *Biostatistics*, vol. 17, no. 1, pp. 29–39, 2016.

15. F. Takens, "Detecting strange attractors in turbulence," in *Dynamical systems and turbulence, Warwick 1980*, pp. 366–381, Springer, 1981.

16. T. Sauer, J. A. Yorke, and M. Casdagli, "Embedology," *Journal of Statistical Physics*, vol. 65, no. 3, pp. 579–616, 1991.

17. J. Huke, "Embedding nonlinear dynamical systems: A guide to takens' theorem," 2006.

18. G. Casella and R. L. Berger, *Statistical inference*, vol. 2. Duxbury Pacific Grove, CA, 2002.

19. M. E. Hughes, L. DiTacchio, K. R. Hayes, C. Vollmers, S. Pulivarthy, J. E. Baggs, S. Panda, and J. B. Hogenesch, "Harmonics of circadian gene transcription in mammals," *PLoS genetics*, vol. 5, no. 4, p. e1000442, 2009.

20. J. Yeung, J. Mermet, C. Jouffe, J. Marquis, A. Charpagne, F. Gachon, and F. Naef, "Transcription factor activity rhythms and tissue-specific chromatin interactions explain circadian gene expression across organs," *Genome research*, vol. 28, no. 2, pp. 182–191, 2018.

21. J. J. Hughey, T. Hastie, and A. J. Butte, "ZeitZeiger: supervised learning for high-dimensional data from an oscillatory system," *Nucleic Acids Research*, p. gkw030, 2016.
